# Effectiveness of vector control methods for the control of cutaneous and visceral leishmaniasis: A meta-review

**DOI:** 10.1371/journal.pntd.0009309

**Published:** 2021-05-13

**Authors:** Carlos Alberto Montenegro Quiñonez, Silvia Runge-Ranzinger, Kazi Mizanur Rahman, Olaf Horstick

**Affiliations:** 1 Heidelberg Institute of Global Health, University of Heidelberg, Heidelberg, Germany; 2 Instituto de Investigaciones, Centro Universitario de Zacapa, Universidad de San Carlos de Guatemala, Zacapa, Guatemala; 3 The University of Sydney, University Centre for Rural Health, Lismore, New South Wales, Australia; Federal University of Ceará, Fortaleza, Brazil, BRAZIL

## Abstract

Elimination of visceral leishmaniasis (VL) in Southeast Asia and global control of cutaneous leishmaniasis (CL) and VL are priorities of the World Health Organization (WHO). But is the existing evidence good enough for public health recommendations? This meta-review summarises the available and new evidence for vector control with the aims of establishing what is known about the value of vector control for the control of CL and VL, establishing gaps in knowledge, and particularly focusing on key recommendations for further scientific work. This meta-review follows the Preferred Reporting Items for Systematic reviews and Meta-Analyses (PRISMA) criteria, including (1) systematic reviews and meta-analyses (SRs/MAs) for (2) vector control methods and strategies and (3) for the control of CL and/or VL. Nine SRs/MAs were included, with different research questions and inclusion/exclusion criteria. The methods analysed for vector control can be broadly classified into (1) indoor residual spraying (IRS); (2) insecticide-treated nets (ITNs; including insecticide-impregnated bednets); (3) insecticide-treated curtains (ITCs; including insecticide-treated house screening); (4) insecticide-treated bedsheets (ITSs) and insecticide-treated fabrics (ITFs; including insecticide-treated clothing) and (5) durable wall lining (treated with insecticides) and other environmental measures to protect the house; (6) control of the reservoir host; and (7) strengthening vector control operations through health education. The existing SRs/MAs include a large variation of different primary studies, even for the same specific research sub-question. Also, the SRs/MAs are outdated, using available information until earlier than 2018 only. Assessing the quality of the SRs/MAs, there is a considerable degree of variation. It is therefore very difficult to summarise the results of the available SRs/MAs, with contradictory results for both vector indices and—if available—human transmission data. Conclusions of this meta-review are that (1) existing SRs/MAs and their results make policy recommendations for evidence-based vector control difficult; (2) further work is needed to establish efficacy and community effectiveness of key vector control methods with specific SRs and MAs (3) including vector and human transmission parameters; and (4) attempting to conclude with recommendations in different transmission scenarios.

## Introduction

Elimination of visceral leishmaniasis (VL) in Southeast Asia and global control of cutaneous leishmaniasis (CL) and VL are priorities of the World Health Organizations’ Department of Control of Neglected Tropical Diseases (WHO NTD). From the 5 elimination strategies for VL, vector control is one of the core ones against both forms of leishmaniasis [1]. However, the effectiveness of vector control for reduction of transmission of both VL and CL is repeatedly under discussion. This has been assessed in numerous reports, including WHO reports, e.g., WHO (2010) [2], but also in comprehensive reviews.

In this sense, Picado and colleagues (2012) [3] presented a review of studies for VL published in the period 2005 to 2010 on the efficacy of different tools to control *Phlebotomus argentipes*. The review indicates that “the current indoor residual spraying (IRS) and novel vector control methods mainly insecticide-treated nets (ITN) have low effectiveness for several reasons. Efforts to improve quality of IRS operations and further research on alternative and integrated vector control methods need to be promoted to reach the VL elimination target by 2015.” However, the review stops short of recommending particular interventions and/or combinations of interventions. In a recent field trial using cluster randomised design, partially unexplored options were also tested for sand fly control [4] in order to strengthen the campaign for elimination, the deadline of which was extended from 2015 to 2020 [5]—no definite recommendations are available at this stage.

Similarly, Kassi and colleagues (2008) [6] conclude in a review for CL that “… it can be seen that many effective interventions exist. Considering the multitude of factors involved in transmission of CL and the various effective control measures tried and tested by investigators, an interdisciplinary approach involving more than one of the above interventions would make sense.” As for VL, the review is not highlighting a particular vector control method or combinations of interventions for CL as most effective and/or recommendable.

However, CL and particularly VL are major public health problems in many countries, WHO reports that “8 countries and territories are endemic for leishmaniasis in 2018. This includes 68 countries that are endemic for both VL and CL, 9 countries that are endemic for VL only and 21 countries that are endemic for CL only” [7]. For VL, the incidence has become very low in Southeast Asian countries, which is related to the Regional Visceral Leishmaniasis Elimination Initiative [8], but in the post-elimination era, it will be a major challenge to sustain elimination, and investments are required for the remaining areas of scientific uncertainty [9]. With this in mind, WHO is making efforts to implement policies and strategies to reduce the disease burden of CL and VL—but is the existing evidence good enough for public health recommendations?

This meta-review follows up on these efforts and aims at facilitating further decision-making processes in 2020, with a process of systematically summarising the available and new evidence for vector control for CL and VL, focusing on existing systematic reviews and meta-analyses (SRs/MAs) and following the Preferred Reporting Items for Systematic reviews and Meta-Analyses (PRISMA) criteria [10]. The key objective is to develop a meta-review including all quality SRs/MAs for the control of

1. CL and VL;
2. through vector control;
3. without geographic restriction; and
4. indexed in English but accepting all other publication languages

with the aims of establishing what is known about the value of vector control for the control of CL and VL, establishing gaps in knowledge, and particularly focusing on key recommendations for further scientific work.

## Methods

### Search strategy, databases, and search terms

This meta-review follows the PRISMA criteria [10]. Literature searches and analysis were developed and carried out through May 31, 2020. Data were extracted from Cochrane Database for Systematic Reviews, Lilacs, PubMed, Wholis, and Google Scholar (the latter was screened for the first 200 hits, since the database is sorted by relevance, 200 hits was established as a suitable number, with no relevant hits encountered towards the end of the search). All full-text assessed articles have been manually searched for additional articles in the reference list. As for grey literature, relevant global guidelines for CL and VL have been identified on WHO Iris: Guidelines were included if published after 2007, when the WHO guidelines committee assumed their work [11] and assuming that global guidelines are using SRs for their recommendations. Relevant SRs have been included.

The inclusion criteria were (1) SRs/MAs for (2) vector control methods and strategies and (3) for the control of CL and/or VL.

Excluded were SRs for clinical picture and treatment, diagnosis, and surveillance.

No restrictions were applied regarding year of publication, geographic area, or publication language; however, the included SRs/MAs needed to be indexed in English, as the search was performed in English.

1. Scientific method of the study: “Systematic review” and/or “Meta-analysis”
2. Disease: “Leishmaniasis” (MeSH term, where applicable), considering that this will always include “Cutaneous leishmaniasis” and “Visceral leishmaniasis.”
  Searches were performed with combinations of the first 2 categories. The search was intentionally kept very broad, and a third category was used with the results obtained from the searches: Articles were retained if they were mentioning the
3. Intervention: “Vector control”, and its variations of different methods.

**Fig 1:**
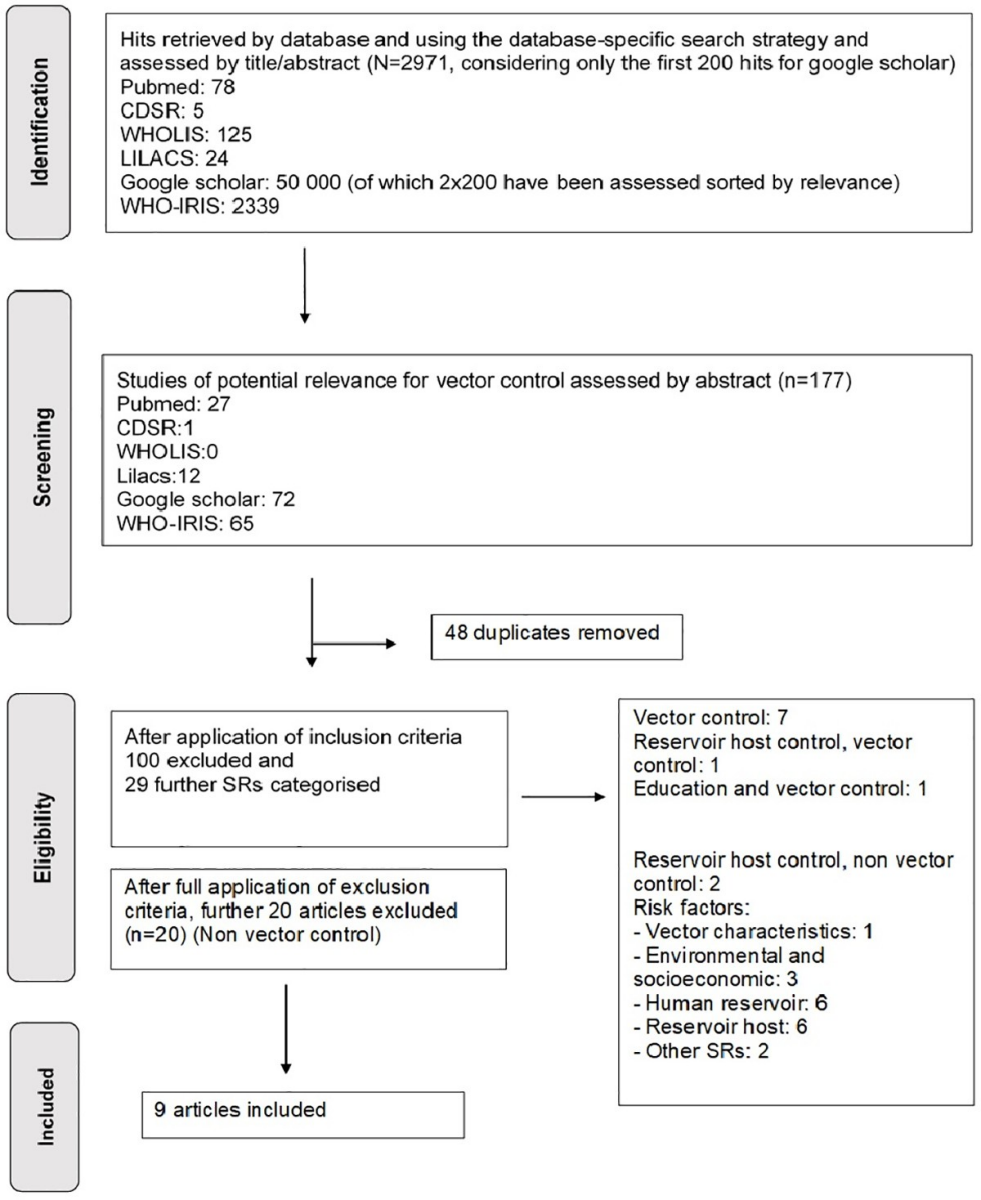
PRISMA flowchart.

Selection of relevant articles was based on study title and abstract and, where needed, by full-text assessment (see Fig 1). The selection and categories of the studies included were initially based on 3 broad categories:

1. SRs or MAs focused on CL vector control, with 1 single intervention only;
2. SRs or MAs focused on VL vector control, with 1 single intervention only; and
3. Combinations of vector control interventions, either with a focus on VL or CL, or combined.

### Quality assessment and assurance

Two data extractors (CAMQ and SRR) independently screened titles and abstracts and applied inclusion and exclusion criteria. In case of disagreement, a third researcher (OH) was involved to reach consensus. Data have been extracted in a predefined data extraction matrix and analysed by author, title, publication date, and study design and main outcomes.

All included articles have been graded for quality by applying the PRISMA checklist [10]. The quality assessment has been used when analysing the results, giving more emphasis on high-quality SRs.

### Data extraction and analysis

The above described data extraction matrix was further developed by including relevant variables such as type of disease, vector control forms, and regions included. All relevant data were extracted into the developed matrix. Evidence tables and recommendations for vector control have been developed and graded for level of evidence as well as strength of effectiveness followed by a gap analysis for further SRs/MAs. The gap analysis included geographical criteria, key vector control interventions, elapsed time since last searches, and quality criteria.

## Results

### Descriptive results

#### Results of searches

A total of 2,971 initial hits were retrieved on the 6 included databases. After assessing by title and abstract, 177 articles were further screened. Moreover, 129 articles were fully assessed, removing 48 duplicates. In total, 29 SRs/MAs remained, of which only 9 dealt with vector control in the broader sense (1. targeting directly the vector; 2. targeting the vector though interventions using the reservoir host; and 3. education related to vector control). All other SRs were concerned about nonvector control issues, e.g., epidemiology of infections, both in humans (6) and in reservoir host (6), nonvector reservoir host control (2) socioeconomic status of cases (3), characteristics of the vector (1); and others (2) (see Fig 1).

A total of 4 SRs/MAs were retrieved on Google Scholar, 3 on PubMed, 1 each on Lilacs, and WHO Iris. In the case of WHO Iris, this database should have WHO guidelines included, and these should have SRs and MAs included for the development of guidelines (see Table 1).

**Table 1:** Evidence table.

SR ID	Papers included/ Inclusion criteria	Countries of study	Quality grading	CL/VL	Results	Results	Results	Results	Results	Results	Results	Conclusions
					**IRS**	**ITNS**	**ITCS**	**ITS and ITF**	**EVM/education**	**Multiple interventions**	**Reservoir control**	
	**Vector Control**											
**González et al. (2015) [17]**	**14 RCTs (8 CL/6 VL)** **Inclusion criteria:** -RCTs -Any intervention that aims to reduce leishmaniasis incidence through vector or reservoir control. -Participants living in leishmaniasis endemic regions	Afghanistan (1) Iran (2) India (1) Bangladesh (1) Colombia (2) Brazil (3) Venezuela (2) India (1) Bangladesh and Nepal (1) India and Nepal (1)	**Quality of included papers:** 5 studies did have a clear baseline measurement The evidence for CL reduction with the individual interventions (ITNs, ITS, ITCs, or IRS) are of moderate or low quality. This means some confidence in these estimates of effect but further research is warranted. The trial that evaluated the protective effect of ITNs against VL had a moderate quality. **PRISMA grading of the SR:** 21/27	**CL**	**IRS versus no intervention on CL** -Two out of 4 cluster RCTs, both from South America evaluated the effect of IRS on vector density. -The trials reported substantial reductions in vectors at the intervention -The 2 trials from South America reported short-term reductions after the intervention but did not provide data to allow to quantify the magnitude or duration of this effect -One cluster RCT from Afghanistan evaluated the effect of IRS on CL incidence. The cumulative analysis of new cases over 15 months showed a marked reduction in clinical cases with IRS	**ITNs versus no intervention or untreated nets on CL** -One of 3 cluster RCTs evaluated the effect of ITNs on vector density -In this study from Iran, the trial authors reported a statistically significant reduction but did not provide data to enable quantification of the magnitude or duration of effect -Two cluster RCTs from Afghanistan and Iran evaluated the effect of ITNs on the incidence of CL. In Afghanistan, ITNs were distributed to all households, and the cumulative analysis of new cases over 15 months showed a marked reduction in CL in areas with ITNs compared to control areas. In Iran, there again appeared to be a large reduction in CL cases. However, the trial authors did not adjust for the cluster design. -In the combined analysis of both trials there was a significant reduction of CL cases.	**ITCs versus untreated curtains or no curtains on CL** -One cluster RCT evaluated the effect of ITCs on vector density -There were no significant differences in mean number of phlebotomine sand flies per house per night between the intervention and control groups before the placement of the curtains. -In the same study, the incidence of clinical cases of CL was 0/1,351 (0%) in the intervention group and 142/1,587 (9%) in the control group. The trial authors reported a cluster adjusted mean difference in CL incidence between the intervention and control areas which is statistically significant.	**ITS (bedsheets) versus no intervention on CL** -A cluster RCT from Afghanistan evaluated the effect of treating bedsheets with permethrin on CL incidence. In the cumulative analysis of new cases over 15 months follow-up, there were substantially fewer in the intervention households. The effect appears to be consistent across age groups **Insecticide-treated uniforms versus no intervention evaluated effects on CL:** -Two individually randomised trials evaluated the effect of impregnating soldiers’ uniforms with permethrin on the incidence of CL. The trials were small and underpowered to confidently detect or exclude effects. However, in one study, the incidence in the control group was 18/143 over 12 weeks (12%), and just 4/143 (3%) in soldiers with impregnated uniforms which did reach standard levels of statistical significance (RR 0.22, 95% CI 0.08 to 0.64).		**Multifaceted intervention versus no intervention evaluated effects on the disease on CL** -A cluster RCT from Colombia evaluated a multifaceted intervention combining ITNs (deltamethrin), personal insect repellent (diethyltoluamide 20%), painting of tree trunks around residences with whitewash, and health education. Over 1 year follow-up there was no statistically significant difference in new cases of CL between intervention and control villages and also no difference in seroconversion.		Using insecticides to reduce phlebotomine sand fly numbers may be effective at reducing the incidence of CL, but there is insufficient evidence from trials to know whether it is better to spray the internal walls of houses or to treat bednets, curtains, bedsheets, or clothing.
				**VL**	**IRS versus no intervention on VL** -No trials evaluated the effects of IRS on VL incidence. However, one trial assessed the effect on seroconversion in a VL endemic area in Brazil and found no statistically significant difference in seroconversion over 18 months post-intervention	**ITNs versus no intervention or untreated nets on VL** -Two out of the 3 cluster RCTs in Asia, evaluated the effect of ITNs on vector density -In Bangladesh, there was a substantial reduction in vector density in the ITN areas for 12 months post-intervention. -In the multicentre trial from Asia, the overall difference between intervention and control sites was not statistically significant. -One additional cluster RCT in India reported a statistically significant reduction in male *P. argentipes* in areas with ITNs compared to untreated nets, but no difference in female *P. argentipes* or other vectors -One cluster RCT evaluated the effect of ITNs on VL in India and Nepal. The overall risk of VL during the 30 months follow-up was 37/9,829 (0.38%) in the intervention group and 40/9,981 (0.40%) in the control group. -In the same trial, there was also no significant difference in the risk of seroconversion in those who had negative results at baseline.		**ITS (bedsheets) versus no intervention on VL** -One cluster RCT in areas of Brazil with VL evaluated the effects of treated sheets and hanging them near the chicken shed. -The trial authors reported short-term reductions in geometric mean phlebotomine sand flies per trap after the intervention, which only differed statistically from control sheds at week 12 post-intervention	**EVM versus no intervention on VL** -The 2 cluster RCTs in areas of Asia with VL evaluated the effect of EVM on vector density -Neither trial found evidence of statistically significant reductions in phlebotomine sand flies compared to no intervention up to 12 months follow-up.	**Multifaceted intervention versus no intervention evaluated effects on the disease on VL** -One additional trial from an area endemic with VL in Brazil evaluated IRS plus culling of infected dogs and found no statistically significant difference in seroconversion over 18 months post-intervention. **IRS versus ITNs, ITCs, or ITS on VL** -Two cluster RCTs in areas of Asia with VL evaluated the comparative effect of IRS and ITNs -In a trial from Bangladesh, India and Nepal, the pooled data with a follow-up at 5 months on trapped phlebotomine sand flies in houses showed that IRS was effective with an average sand fly reduction of about 50%, but the ITNs had very little effect. -In one trial from Bangladesh, both interventions were associated with a decrease in sand fly density at 5 months. -A cluster RCT in areas of Brazil with VL, included a comparison of IRS with insecticide-impregnated cotton sheets or blankets. Following IRS intervention, *Lu. longipalpis* abundance fell by only 45% versus 90% after ITS intervention on week 12 post-intervention. IRS versus ITNs, ITCs, or ITS on VL -In the multi-arm cluster RCT from Afghanistan, the differences in CL incidence between clusters allocated to IRS, ITNs or ITS did not reach standard levels of statistical significant differences among interventions over 15 months. IRS versus EVM on VL -Two cluster RCTs in areas of Asia with VL also evaluated the effect of IRS versus EVM on vector density, the pooled data in both trials showed that EVM had no or very little effect on total sand fly density. **ITNs versus EVM on VL** -Two cluster RCTs in areas of Asia with VL also compared long-lasting ITN with EVM, only ITNs had an important effect on the average reduction of phlebotomine sand flies at 5 months. **Reservoir control versus IRS on VL:** -A cluster RCT based in a VL-endemic area in Brazil, evaluated the effects of insecticide spraying of animal pens, and reservoir control (eliminating infected dogs) on seroconversion, using IRS of houses alone as the control group. IRS of houses and elimination of infected dogs appeared to reduce seroconversion compared to IRS alone. However, this effect was not seen in a similar comparison of IRS of houses and animal pens plus elimination of infected dogs versus IRS alone.	**Reservoir control versus no intervention on VL:** -No trials evaluated the effect of reservoir control on clinical disease but one trial from an area endemic with VL in Brazil found a 38% reduction in seroconversion over 18 months post-elimination of infected dogs (RR 0.62, 95% CI 0.42 to 0.91, 1 trial, 376 participants in 20 clusters)	There is currently no evidence that these measures are effective or not in reducing VL incidence.
				**CL/VL**	**IRS versus no intervention (no clear division on VL or CL)** -Two out of 4 cluster RCTs, from Asia, evaluated the effect of IRS on vector density -The trials reported substantial reductions in vectors at the intervention sites -Large reductions were seen with IRS compared to control areas in these 2 trials from Asia							Policy decisions should consider local sand fly epidemiology and behaviour, as well as the diversity of transmission scenarios (including vector and animal or human reservoirs) when designing and implementing leishmaniasis control programmes.
**Wilson et al. (2014) [15]**	**21** **RCTS** **NRTS** **Rotational studies from which only 9 were on CL and 3 on VL** **Inclusion criteria:** -Studies were included if they compared the efficacy of ITNs, ITCs or ITS versus no intervention (control group) in disease endemic areas -Studies using hand-impregnated nets or factory-manufactured LLINs were included -Studies assessed the effect of the intervention on either (i) clinical outcomes; or (ii) entomological outcomes	12 studies on CL and VL: Iran (3) Afghanistan (1) Colombia (2) Turkey (1) Burkina Faso (1) Sudan (1) Venezuela (1) India, Bangladesh, and Nepal (1) India and Nepal (1)	**Quality of included papers:** Of the 21 studies identified, 15 were deemed to be at low risk of bias, 3 at medium risk, and 3 at high risk of bias. Twelve studies were deemed to be of high quality, 3 medium quality, and 6 low quality. **PRISMA grading of the SR:** 27/27	CL		**Efficacy of ITNs against CL:** -A total of 6 studies assessing the efficacy of ITNs against cutaneous leishmaniasis were identified. -Preintervention incidence of cutaneous leishmaniasis was comparable in intervention and control groups in the 3 studies. -Random effect MA indicated a PE of ITNs against cutaneous leishmaniasis of 77%. -Another study reported a significant reduction in incidence of cutaneous leishmaniasis in ITN clusters. -Studies assessing the efficacy of ITNs reported mixed results in terms of effect on sand fly density ranging from a relative increase of 49% to a relative reduction of 96%. -One study reported a highly significant PE against cutaneous leishmaniasis in Iran; no effect on the mean number of Phlebotomus sergenti captured per month was detected in this study. -Another study reported a beneficial effect of ITNs on clinical disease in Turkey and a percentage increase in vector density relative to the control group was documented. -One study demonstrated a high PE against cutaneous leishmaniasis of 93% in Venezuela	**Efficacy of ITCs against CL:** -Studies that measured the entomological effect of ITCs demonstrated a high percentage reduction in vector density of 54%, 87%, and 98%. However, the 98% reduction was observed in a study that was deemed to be of low quality.			**Efficacy of ITCs and ITS against CL:** -A study which assessed the efficacy of ITCs and ITS against cutaneous leishmaniasis in Iran reported a PE of 16%. This study was deemed to be of low quality due to the study design and high risk of bias.		-MA of clinical data could only be performed for 4 cutaneous leishmaniasis studies which together showed a protective efficacy of ITNs of 77% (95% CI: 39%-91%). -Studies of ITC and ITS against cutaneous leishmaniasis also reported significant reductions in disease incidence. -High protective efficacy of ITNs against cutaneous leishmaniasis, which suggests that there may be considerable collateral benefits of ITN roll out where cutaneous leishmaniasis and malaria are coendemic -There is also good evidence of the efficacy of ITC and ITS against cutaneous leishmaniasis -Non-malaria endemic countries where cutaneous leishmaniasis is prevalent should consider rolling out ITNs as part of control efforts
				VL		**Efficacy of ITNs against VL:** -Three studies assessing the efficacy of ITNs on visceral leishmaniasis were identified. -One study did not show a significant effect on incident *Leishmania donovani* infections or incident cases of visceral leishmaniasis. -In India and Nepal, the same study, however, did appear to show an effect on vector density with a relative reduction in the mean number of *P. argentipes* females. -Two studies conducted in Sudan and Bangladesh, India, and Nepal demonstrated a 100% and 35% reduction in vector density, respectively. -No studies were identified which assessed the efficacy of ITCs or ITS against visceral leishmaniasis.						-Clinical evidence from one study suggested that ITNs were not effective against visceral leishmaniasis. However, in this study it is suggested that *L*. *donovani* transmission may have been occurring outside the home where ITNs would have little impact on preventing sand fly–human contact.
				**CL/VL**								-The efficacy of ITNs in preventing leishmaniasis transmission is dependent on a number of key variables related to vector biology, type of nets, and human behaviour. -In general, where transmission is occurring inside the home or where vectors rest indoors, the authors would expect ITNs, ITCs, or ITS to have a beneficial effect, irrespective of whether the vectors are transmitting cutaneous or visceral leishmaniasis.
**Romero and Boelaert (2010) [12]**	**14** **specific on VL control** **Inclusion criteria:** original studies evaluating human diagnosis; clinical trials including uncontrolled and retrospective studies with description of human treatments; original studies evaluating any diagnostic test for canine leishmaniasis; Field trials of control measures evaluating at least 1 control measure (canine culling, impregnated dog collars, canine vaccination, insecticide spraying, insecticide-treated bednets, environmental management)	Brazil (13) Venezuela (1)	**Quality of included papers:** The quality of the included paper was affected by some limitations: -Low sample size -Loss of follow-up -Ineffective interventions in some studies -Areas submitted to heterogeneous follow-up -No control arms -Non-comparable baseline prevalence **PRISMA grading of the SR:** 15/27	**VL**	**IRS on VL:** The studies that evaluated IRS and fogging around the houses, a significant decrease of sand fly abundance was observed, with a residual effect of indoor spraying, that usual lasted 3 months. The specific construction style of the houses lowered the external validity of the study.	**ITNS on VL:** One study reported the barrier effect, the 24-hour mortality rate and the human landing rates of *Lutzomiya longipalpis* in households using deltamethrin-impregnated bednets compared others using untreated bednets. The study described a 39% increase in barrier capacity of the impregnated bednets, 80% reduction in sand fly landing rates on humans and 98% increase in the 24-hour sand fly mortality rates. Despite the limitations (a small number of observations, a short period of exposure and without the measure of the residual effect), this intervention should be explored further because it could bring an additional benefit in areas where malaria is also endemic.				**Multiple interventions on VL:** Studies that reported the impact of combined intervention, usual consisted in the combination of human VL case treatment, culling of seropositive dogs and insecticide spraying, one study reported the disappearance of human symptomatic cases after 15 years of application of this strategy. Another observed that the intensity of the application of control measures correlated with human VL incidence, the coverage of canine surveys, the number of canine surveyed, and the number of buildings submitted to insecticide spraying. Also, in combined interventions, the results of one study indicated a positive effect of canine removal on incidence of leishmanial infection in men but surprisingly, the combination of dog culling plus outdoor spraying of peridomestic animal shelters failed to demonstrate any effect. Another study reported that although a lower incidence was observed in the groups submitted to combined interventions and that reduction was more intense after 2 years, the study failed to detect statistically significant differences. Another study reported the effect of application of a 65% permethrin spot-on formulation on canine VL infection and sand fly abundance. A decrease of canine VL prevalence was observed in the intervention area compared with increased prevalence in the control area. No effect was observed on sand fly population.	**Reservoir control on VL:** The studies that reported dog screening and culling, usually screened by ELISA and IFAT and reported a reduction of seroconversion rate in dogs but without a significative difference. It was also observed that by using method ELISA, the reduction of canine seroprevalence was higher, probably due faster dog removal plus higher sensitivity of the ELISA test. When continuing with seropositive dog removal, a significant reduction of dog seroconversion rate in the intervention areas as compared to control was observed, and a significantly lower number of VL cases reported to health facilities in the intervention area. In some cases, the incidence of canine infection remained stable through 2.5 years of observation under this strategy. Dog migration was usually not observed, but a study highlighted the challenges posed by dog migration for any control programmes dealing with the canine reservoir. A Phase III vaccine field trial in seronegative dogs screened with IFAT and FML-ELISA and exposed to fucose-mannose-ligand vaccine in 3 subcutaneous doses at 21-day intervals, showed a significant difference in the 3 endpoints observed during the trial. The overall efficacy to prevent symptomatic VL disease was 75%. In a study that reported a controlled field trial to evaluate the effectiveness of insecticide impregnated collars to prevent infection detected through serological tests or DNA detection, the authors failed to detect a significant difference between groups in the incidence of new infections but they demonstrated a significant reduction of antibody titers in the collar protected dogs.	-Canine culling seems to be the least acceptable intervention at community level and has low efficiency due to high replacement rate of eliminated dogs with susceptible puppies. Vector control interventions are better accepted by the affected populations and mathematical models suggested encouraging efficacy, but they need further study. Better knowledge of vector seasonality and behaviour is required for proper timing of these interventions. The current evidence indicates that spatial fogging is useless and that the residual effect of house wall spraying is very short. Insecticide impregnated collars seem to have a longer residual effect and theoretical advantages over the other methods and should be studied in larger and well-designed controlled trials. The potential emergence of resistance to insecticides should also be considered for the long-term planning of any vector control intervention. There are a few well-designed intervention studies for control of vectors or canine reservoirs. Elimination of zoonotic VL in the Americas does not seem a realistic goal at this point given the lack of political commitment, gaps in scientific knowledge, and the weakness of case management and surveillance systems. Research priorities and current strategies should be reviewed with the aim of achieving better VL control.
**Stockdale and Newton (2013) [14]**	**Of 84 studies, only 35% measured Leishmania infection in humans, 13 on CL, 5 on VL, and 1 on both** **Inclusion criteria:** Only RCTs and controlled trials were included. Any form of leishmaniasis, and any preventative method relating to vector control, human, or animal reservoir control were included. The search was not limited by date of publication and includes everything identified up until October 2012.	Iran (6) Brazil (4) Peru (1) Afghanistan (2) India and Nepal (1) Syria (2) Venezuela (1) Colombia (2)	**Quality of included papers:** Lack of generalizability No reference to randomisation of intervention and control areas is made Absence of data pre and post-intervention Potential bias in case detection affected the quality of the papers **PRISMA grading of the SR:** 16/27	CL	Of the 22 studies included in the vector control section, only 4 investigated human-specific outcome measures following interventions directed at controlling the vector population. All of these involved insecticides spraying of houses and other buildings. **IRS on CL:** -A study used semi-randomisation of houses in 3 villages allocated to either intervention (sprayed) or control (unsprayed) arms. The group reported a significant difference in CL cases over 2 years between the control houses and the intervention areas. -One study conducted a large cluster randomised trial of management of CL in Afghanistan, with 4 arms. Two of the 4 studies using IRS, use robust diagnostic methodologies (serology) and show no difference between intervention and control, and two use less robust methods of diagnosis and show a statistically significant effect. Based on this evidence, there is not enough information to determine if insecticide spraying is advantageous in reducing the burden of disease in humans.	**ITNS on CL:** The remaining 5 net studies all report a statistically significant reduction of cases of CL by using ITNs. A general criticism regarding these five is that, except one, the others all used self-reporting as the method of diagnosis which is at high risk of reporting bias.	**ITCS on CL:** One of the 4 studies that used insecticide-impregnated fabrics, used impregnated and nonimpregnated curtains placed on windows, reporting a statistically significant decrease in numbers of cases of Leishmania infection between intervention and control groups	**ITF on CL:** Four studies used insecticide-impregnated fabrics in an attempt to reduce the burden of disease caused by leishmaniasis. Of these, 3 reported a statistically significant decrease in numbers of cases of Leishmania infection between intervention and control groups, and one did not (intervention with soldier’s uniforms). Two used permethrin-impregnated clothing for soldiers and one used permethrin-treated bed sheets.	**EVM:** None of the studies measuring environmental management study human-specific outcomes.	In all of the animal culling studies reviewed here, as well as all but one insecticide study, the report makes no reference to randomisation of areas. Only 1 study (Ershadi et al.) do however state that pre-intervention rates of infection were similar between the 2 areas investigated, but no data to support this assertion were presented. Four studies included multiple interventions which were applied to the population of interest: **Multiple interventions on CL:** One study evaluated the use of pyrethroid impregnated bednets, window and door curtains, and health education. The group reported a statistically significant decrease in parasitologically confirmed cases of CL in 1 year, in households using the insecticide-treated bednets and curtains in 3 randomly selected neighbourhoods in Iran which were matched for pre-intervention prevalence of CL.	Four of the 34 studies evaluating reservoir control, investigated human-specific outcomes as a result of interventions directed at animal reservoir control. Three of the 4 studies reported statistically significant reductions in human Leishmania infection between the intervention and control areas. Two of these were animal elimination programmes, and one was an insecticide-impregnated dog collar study.	
				**VL**	**IRS on VL:** -One study evaluated VL in children under 12 years in 3 control and 3 intervention areas subject to intradomiciliary IRS every 6 months. No reference is made to randomisation of areas although the authors do state that background infection rates were approximately equal in all 3 areas. -Another study used random allocation of areas into 4 interventions in order to study VL in the population living in each area. Background VL prevalence was 42% in the intervention areas and 31% in the control area					**Multiple interventions on VL:** Two studies investigated human VL infection by serological methods following the concurrent spraying of insecticide and dog culling. One study claim significance, whereas the other do not. Neither study reports P values. The last study used multiple interventions consisting of treated bednets, modification of sand fly resting areas, and health education.	**Reservoir control on VL:** Studies that reported dog elimination as an intervention for VL, did not found a significant difference in human seropositivity. One study indicated that, although the major mode of transmission in Brazil is thought to be zoonotic (with dogs as the major reservoir), the authors hypothesised a greater than previously thought role for humans as the significant reservoir for VL. Another study had several limitations in measuring the results, for example not indicating if the intervention included domestic and feral dogs. Or these was a lack compromise in the follow-up of the populations intervened, such as rodents or dogs. An additional study which investigated human infection used an insecticide-impregnated dog collar as intervention. Although the intervention is associated with a statistically significant decrease in child VL prevalence during the 1 year follow-up, the actual numbers of seroconversions are low—17 in the 9 intervention villages, and 26 in the 9 control villages.	
				**CL/VL**		**ITNS on both CL and VL** For human reservoir control, 7 studies using ITNs measured a human-specific outcome. Two studies used deltamethrin-impregnated bednets. Neither group reported any difference in cases of CL or VL between the treated nets and either untreated nets or existing intervention						This review highlights an absence of research measuring human-specific outcomes (35% of the total) across all intervention categories. The apparent inability of study findings to be generalizable across different geographic locations points towards gaps in knowledge regarding the biology of transmission of Leishmania in different settings. More research is needed which investigates human infection as the primary outcome measure as opposed to intermediate surrogate markers, with a focus on developing a human vaccine. Because of the small number of studies and the potential for bias, limited conclusions can be drawn as to the efficacy of interventions aimed at reducing animal reservoir infection with Leishmania on reducing disease burden in humans. In order to address this gap in knowledge, more and larger studies investigating human infection are needed. In terms of generalizability, indoor insecticide spraying programmes are only useful where the sand fly is likely to come into contact with the walls that are actually sprayed, which necessitates endophagic or endophilic (either biting or resting indoors) sand fly species. It is not clear from any of the papers reviewed here that the authors took this into consideration. Further research would be needed in order to characterise the feeding, resting and breeding habits of different sand fly species in each endemic area, prior to implementation of a preventative intervention. In addition, more studies are needed which measure human infection after vector control interventions. Bednet studies have been used extensively in malaria prevention programmes to great effect and the robust cluster randomised study designs appear to have been largely well replicated in leishmaniasis studies. However, as evidenced by the highly heterogeneous studies reviewed here, there are inconsistencies and lack of standardisation in study design which has resulted in groups reporting data which cannot be combined to produce reliable summary estimates. The evidence suggests that use of insecticide impregnated fabric, whether curtains or fabrics worn next to the skin is associated with a decrease in Leishmania infection. The generalizability of curtain use would depend on the feeding characteristics of the sand fly vector specific to the area of interest and may only have an effect in areas where transmission occurs indoors. The uptake of using insecticide-impregnated fabrics next to the skin might be problematic if those insecticides cause skin irritation. Based on our current lack of understanding of the transmission of Leishmania, it seems salient to focus scant resources on prevention of human infection, as opposed to interventions which attempt to address upstream risk.
**Kappagoda and Ioannidis (2014) [16]**	**258 RCTs: 46 RCTs in Leishmaniasis, from which 17 were interventions targeting vectors (CL and VL)** **Inclusion criteria:** RCTs published on or before December 31, 2012 that addressed the prevention or control of 16 NTDs (including leishmaniasis). sought additional trials by reviewing own literature collections, English-language SRs, MAs, and Cochrane reviews, and the references of eligible publications identified. A separated search for SR and MA	Not discussed	**Quality of included papers:** not discussed **PRISMA grading of the SR:** 12/27	**CL/VL**						**Both VL and CL:** SRs evaluated the effectiveness of interventions of Leishmaniasis and found that insecticide spraying and dog culling (combined or not) were likely to be ineffective.	**Both VL and CL:** In addition, more than 1 SR assessed the effectiveness of dog culling, which found to be consistently likely to be ineffective.	Future MAs would be made easier by standardising the design of trials on vector control strategies, including habitat modification, the use of insecticide-impregnated materials, and spraying for dengue and leishmaniasis. Furthermore, most vector control trials for leishmaniasis and dengue did not consider human disease as an outcome and there is a need for more research on the relationship between vector control and human disease to justify such interventions. The most studied diseases were geohelminth infection (51 RCTs) and leishmaniasis (46 RCTs). Vaccines, chemoprophylaxis and interventions targeting insect vectors were evaluated in 113, 99 and 39 RCTs, respectively. Few addressed how best to deliver preventive chemotherapy, such as the choice of dosing interval (10) or target population (4), the population coverage needed to reduce transmission (2) or the method of drug distribution (1). Thirty-one publications containing 32 SRs (16 with and 16 without MAs) were found on American trypanosomiasis, dengue, geohelminths, leishmaniasis, leprosy, lymphatic filariasis, onchocerciasis, schistosomiasis, or trachoma. Together, they included only 79 of the 258 published RCTs (30.6%). Of 36 interventions assessed, 8 were judged effective in more than 1 review. Conclusion: Few RCTs on the prevention or control of the principal NTDs were found. Trials on how best to deliver preventive chemotherapy were particularly rare.
**Horstick and Runge-Ranzinger (2018) [18]**	**32 studies from which 13 on Leishmaniasis either RCTs or cRCTs (6 on VL and 7 on CL)** **Inclusion criteria:** Studies reporting vector control interventions in and around a house or dwelling; and use of insecticides as sprays on netting or screens, and any method to control larval breeding in water containers in and around the home	Three of 13 studies were done in Latin America (1 Brazil and 2 Venezuela) and the remaining 10 were done in Asia or the Middle East (Afghanistan, Bangladesh, India, Iran, and Syria) or in several countries, for example, India and Nepal or India, Nepal, and Bangladesh	**Quality of included papers:** Four leishmaniasis studies were RCTs and four were cRCTs. Of the remaining leishmaniasis studies, one study was a randomised controlled intervention trial and another study was a randomised community intervention trial (RCT), whereas 3 studies had a matched paired cRCT study design (cRCTs). **PRISMA grading of the SR:** 24/27	**CL/VL**					EVM on both VL and CL: Modifications to the structure of homes (e.g., wall plastering) had no impact on the control of vectors. Protection of the house and its surroundings might affect the transmission of several diseases.	On both VL and CL: The most effective interventions affecting vector indices for multiple diseases were found to be intradomiciliary residual spraying, ITMs (especially ITNs or ITCs), and treatment of larval habitats with biological and chemical methods. Waste management and clean-up campaigns reduce vector populations, although to a lesser extent than other interventions and not consistently. The most effective interventions should be prioritised when vector control programmes are designed; however, the quality of delivery (i.e., coverage and reapplication) of interventions is a crucial factor to ensure their effectiveness. Additional randomised trials that assess the measures of human disease and eventually target several diseases with a combination of interventions that protect the household and its inhabitants against multiple vectors, are needed to inform global policy in this area. Studies of leishmaniasis included 39 houses to 3,000 houses with a follow-up of 9 weeks to 24 months		Studies are needed that target several diseases with a combination of interventions and focus on protecting the household against multiple vectors, and thus household members, to provide further evidence for the pressing issue of vector control, especially in dense urban environments. Study design and conduct is a further issue, with very few high-quality studies available. One suggestion is to develop best practice study design, with RCTs and cRCTs following quality criteria on size, study time, and outcomes, including human disease measures as outcomes.
**SR ID**	**Papers included/** **Inclusion criteria**	**Countries of study**	**Quality grading**	**CL/VL**	**Results**	**Results**	**Results**	**Results**	**Results**	**Results**	**Results**	**Conclusions**
					**IRS**	**ITNS**	**ITCS**	**ITS and ITF**	**EVM/education**	**Multiple interventions**	**Reservoir control**	
	**Indirectly targeting vectors, housing**											
**Calderon-Anyosa et al. (2018) [20]**	31 studies from which: 7 on CL and 15 on VL **Inclusion criteria:** Studies that assessed house architecture association (e.g., wall, roof, and floor materials) or the effect of an architectural intervention on either clinical outcomes (prevalence or incidence of cases) and/or entomological outcomes (vector density or mortality) of leishmaniasis were included. No restrictions were applied.	Fifteen of the 31 selected studies were conducted in Asia (9/15 in India, Nepal, and Afghanistan), 11 in Latin America (Brazil, Colombia, Argentina, and Ecuador) and 5 in Africa (Ethiopia and Kenya)	**Quality of included papers:** Not specified **PRISMA grading of the SR:** 20/27	**CL/VL**	**On both VL and CL:** Intervention studies: 4/8 studies evaluated the effect of insecticidal spray on different wall materials, measuring sand fly density captured by light traps (1/4) and sand fly mortality by wall bioassay (3/4). 1/4 study evaluated sand fly density, finding differences associated with housing quality and vector densities, in some cases the number was higher even after insecticide thermal fogging. From the 3/4 studies, one evaluated fogging on cement wall versus oil-painted wall, finding no significant differences in sand fly mortality at 7 or 125 days after fogging, whereas it was significantly higher in oil-painted wall at 69 days. Another study matched houses according to their structure and were randomly assigned to spray treatment or control, finding that sand fly mortality decreased progressively on wood and cement surfaces after 63 days compared with a more rapid decrease on mud and straw walls. The third study evaluated spray on the external and internal surfaces of 3 types of walls, finding that mortality rates were similar, whatever the type of wall since the fourth month.				**EVM on both VL and CL:** This SR found that mud walls with cracks and holes, damp, and dark houses were risk factors for transmission of leishmaniasis. These characteristics create favourable conditions for sand fly breeding and resting as sand flies prefer humidity, warmth, and protection from sunlight during the day. A total of 18/23 studies found significant association between housing characteristics (e.g., walls, roof, floors, or windows) and leishmaniasis infection or sand fly density. Moreover, 16/18 studies found an association between leishmaniasis and wall type. In 15/16 studies found an association with clinical leishmaniasis: 5/ 15 with CL cases; 10/15 were associated with VL cases. A total of 4/8 intervention studies evaluated housing characteristics and home improvement against sand fly density captured by light traps. One experimental study evaluated the characteristics of chicken sheds against sand fly densities finding a significantly higher number of sand flies in open sheds. The 3 remaining studies evaluated the effect of plastering and closing crevices against sand fly densities. One study found no significant difference in sand fly density, whereas the other two found a decrease in sand fly density after the intervention.			Housing interventions might be a promising research area with a special focus on education as individual and collective protection for the effective control of leishmaniasis. Authors found that leishmaniasis is a multifactorial problem, where housing characteristics may play a key role. These characteristics have been demonstrated to be independent risk factors for this disease, but there are still major research gaps. Further studies involving house characteristics are needed; these studies should involve multidisciplinary teams, including healthcare professionals, architects, and engineers to identify risk and to develop new methods of construction using materials accessible in rural areas. Likewise, future interventions should be developed with participation of the community with emphasis on health education as well as housing improvement as individual and collective protection for the effective control of leishmaniasis.
**SR ID**	**Papers included/** **Inclusion criteria**	**Countries of study**	**Quality grading**	**CL/VL**	**Results**	**Results**	**Results**	**Results**	**Results**	**Results**	**Results**	**Conclusions**
					**IRS**	**ITNS**	**ITCS**	**ITS and ITF**	**EVM/education**	**Multiple interventions**	**Reservoir control**	
	**Indirectly targeting vectors, education**											
**de Sousa et al. (2015) [13]**	6 studies (5 Interventions studies, 1 survey), 4 on CL and 2 on VL **Inclusion criteria:** Studies conducted in South America. No restrictions regarding the language of publications, study designs, and sociodemographic characteristics of the target population. Studies were considered for analysis when they included at least 2 of 4 health education categories specified in the text.	Brazil (4x) Colombia and Peru	**Quality of included papers:** Not specified **PRISMA grading of the SR:** 21/27	**CL/VL**					**Education on both VL and CL:** Five studies evaluated the influence of educational materials showing an improvement or reinforcing the importance of educational activities to improve access to knowledge by the population. One study showed the actions of local social representations as effective instruments of information and prevention of leishmaniasis. Also including guidance to the public on the use of screens and mosquito nets impregnated with insecticide. One study found that although 94% of participants knew leishmaniasis as a skin disease, with ulcers or blemishes, only 35% associated the disease with the bite of an infected “mosquito” and only 10% used the appropriate drug treatment.			Leishmaniasis control programmes should be appropriate to the social and cultural realities where they will be applied. All strata and possible segments of the population where the disease is present should be targeted. Health perception of diversified individuals and populations leads to a need and desire for care and is essential at this point. Thus, health education on leishmaniasis should lead to changes in attitudes and habits, from a critical reflective position, allowing the necessary epidemiological transformations and not just be limited to the dissemination of information. Studies on health education in leishmaniasis, in Brazil as well as in other South American countries, should be encouraged because of the wide dispersion and great impact of these diseases in the affected populations. Educational interventions on health occupy an important place regarding the control of neglected diseases because they interfere with several epidemiological components of the disease, presenting potential for transformation.
**SR ID**	**Papers included/** **Inclusion criteria**	**Countries of study**	**Quality grading**	**CL/VL**	**Results**	**Results**	**Results**	**Results**	**Results**	**Results**	**Results**	**Conclusions**
					**IRS**	**ITNS**	**ITCS**	**ITS and ITF**	**EVM/education**	**Multiple interventions**	**Reservoir control**	
	**Reservoir host control (Vector control)**											
**Wylie et al. (2014) [19]**	11 studies all on VL **Inclusion criteria:** studies that investigated prophylactic control measures for naturally occurring *Leishmania infantum* infection on parasite load, humoral (serology) or cellular immunity, infectivity, death, clinical disease and/or adverse effects in dogs; studies that included dogs susceptible to naturally occurring *L*. *infantum* infection but noninfected at the start of the study; serology must have been undertaken as a minimum technique to establish noninfection	Brazil (1) Greece (1) Iran (1) Italy (5) Spain (2) Tunesia (1)	**Quality of included papers:** All of the studies were considered to be at a high risk of methodological shortcomings, with the exception of one spot-on study which was considered to be at an unclear risk of methodological shortcomings. **PRISMA grading of the SR:** 24/27	VL							**Reservoir control on VL:** Use of insecticide-treated dog collars (4 studies including NRCTs and a matched-cluster RCT) and a combination of dog collars and spot-on insecticides treatments (1 NRCT study): There was a statistically significant protective effect for collars for the primary outcome of the overall proportion of dogs infected with L. infantum based on serology or parasite detection for 4 of the 5 studies, with each study presenting an OR<1.00 (range 0.10–0.45) and an ARR>0.00 (range 0.04–0.34); based on IFAT serology only (Study B (a), C and H) and DAT serology (Study D). There was a non-significant result for testing on ELISA and IFAT. Use of spot-on insecticides treatments (3 studies, including NRCTs and RCTs): There was a statistically significant protective effect for spot-ons for the overall proportion of dogs infected with *L. infantum* based on serology or parasite detection, for 4 of the studies (OR range 0.01–0.14) and an ARR > 0.00 (range 0.10–0.37) including the IFAT serology for 65% permethrin (Study B (b)), IFAT serology for 10% imidacloprid and 50% permethrin (Study J), and both doses of 10% imidacloprid and 50% permethrin based on positive immunochromatographic rapid tests or PCR/cytology (Study I (a and b)). There was no significant difference for Study E. Use of prophylactic medications (3 studies, including RCTs): Two studies on prophylactic medications investigated domperidone liquid solution and one investigated allopurinol capsules There was a statistically significant protective effect for prophylactic medication for the overall proportion of dogs infected with *L. infantum* based on serology or parasite detection, for one study based on IFAT serology for domperidone. Studies G and F found that for every 100 dogs prophylactically medicated with 0.5 mg/kg/day domperidone 37 and 6 cases of infection with *L. infantum* based on serology or parasite detection would be averted. There was no significant difference for Study K.	There are studies to support the use of control measures to prevent *L*. *infantum* infection in both endemic and non-endemic areas, in particular deltamethrin collars, 65% permethrin, 10% imidacloprid with 50% permethrin spot-ons and domperidone prophylactic medication. However, the risk of methodological shortcomings with the publications on these interventions needs to be considered. Well-designed, adequately powered RCTs are needed to determine whether using control measures for CanL confers prophylactic benefits.

CL, cutaneous leishmaniasis; cRCT, cluster randomised controlled trial; EVM, environmental modification; IRS, indoor residual spraying; ITC, insecticide-treated curtain; ITN, insecticide-treated net; ITS, insecticide-treated bedsheet; MA, meta-analysis; NRCT, non-randomised clinical trial; NRT, non-randomised trial; PE, protective efficacy; PRISMA, Preferred Reporting Items for Systematic reviews and Meta-Analyses; RCT, randomised controlled trial; RR, risk ratio; SR, systematic review; VL, visceral leishmaniasis.

#### Time and geographical clustering of SRs/MAs

All included SRs/MAs were published between 2010 and 2018. All SRs/MAs were published by different groups of authors and focusing mostly on the global situation of both CL and VL. Romero and Boelaert (2010) [12] and de Sousa and colleagues (2015) [13] focused on Latin America only.

#### Methods applied by the SRs/MAs

All SRs followed the PRISMA guidelines [10], only 1 article was not labelled an SR, but followed predefined criteria, as prescribed by PRISMA [14]. Only 1 article included an MA [15], not surprisingly, since the studies included in the SRs are very heterogenous, with different interventions and outcome measures.

#### Focus of the SRs/MAs

The SRs/MAs included focused on CL and VL from a different viewpoint, assessing vector control from topics that range solely on CL and VL to reservoir host control for VL and health education (see Table 2).

**Table 2:** Overview of the different topics that the included SRs/MAs focused on.

Topic	SRs/MAs	Additional focus
CL/VL	Stockdale and Newton (2013) [14]	
	González et al. (2015) [17]	
	Calderon-Anyosa et al. (2018) [20]	Housing interventions
VL in Latin America	Romero and Boelaert (2010) [12]	
Vector-borne diseases in general (including CL/VL and not including malaria)	Kappagoda and Ioannidis (2014) [16]	
	Wilson et al. (2014) [15]	Only ITNs and ITCs
	Horstick and Runge-Ranzinger (2018) [18]	Interventions for protection of human dwellings
Reservoir host control for VL	Wylie et al. (2014) [19]	
Health education for CL and VL, in Latin America	de Sousa et al. (2015) [13]	

CL, cutaneous leishmaniasis; ITC, insecticide-treated curtain; ITN, insecticide-treated net; MA, meta-analysis; SR, systematic review; VL, visceral leishmaniasis.

This overview shows that comparability between the existing SRs/MAs is difficult, since most of the SRs and MAs have a different focus.

Also, it shows that the initially conceptualised categories for classification—as specified in the Methods section (1. focus on CL vector control, with 1 single intervention only; 2. VL vector control, with 1 single intervention only; and 3. combinations of vector control interventions, either with a focus on VL or CL, or combined) cannot be applied, since the research questions differ (see Table 3).

**Table 3:** Primary evidence used in the included articles.

		SRs and key focus								
		González et al. (2015) [17]	Wilson et al. (2014) [15]	Romero and Boelaert (2010) [12]	Stockdale and Newton (2013) [14]	Kappagoda and Ioannidis (2014) [16]	Horstick and Runge-Ranzinger (2018) [18]	Calderon-Anyosa et al. (2018) [20]	de Sousa et al. (2015) [13]	Wylie et al. (2014) [19]
		Vector and reservoir control	ITN, ITC, and ITM on vector-borne disease other than malaria	VL in Latin America	Prevention of Leishmaniasis	Prevention of NTDs	Protection the house against Chagas, dengue, leishmaniasis, and lymphatic filariasis	Housing and Leishmaniasis	Health education and Leishmaniasis in South America	Controlling canine Leishmanisasis with topical insecticies and medication
**CL**	**IRS**									
	Chaves LF, Calzada JE, Rigg C, Valderrama A, Gottdenker NL, Saldaña A. Leishmaniasis sandfly vector density reduction is less marked in destitute housing after insecticide thermal fogging. Parasit Vectors. 2013;6:164.							X		
	Davies CR, Llanos-Cuentas E, Campos P, Monge J, Leon E, Canales J. Spraying houses in the Peruvian Andes with lambda-cyhalothrin protects residents against cutaneous leishmaniasis. Trans R Soc Trop Med Hyg. 2000;94(6):631–636.				X					
	Feliciangeli MD, Mazzarri MB, Campbell-Lendrum D, Maroli M, Maingon R. Cutaneous leishmaniasis vector control perspectives using lambdacyhelothrin residual house spraying in El Ingenio, Miranda State, Venezuela. Trans R Soc Trop Med Hyg. 2003;97(6):641–6.	X					X	X		
	**ITNs (including insecticide-impregnated bednets)**									
	Alexander B, Usma MC, Cadena H, Quesada BL, Solarte Y, Roa W, et al. Evaluation of deltamethrin-impregnated bednets and curtains against phlebotomine sandflies in Valle del Cauca, Colombia. Med Vet Entomol. 1995;9(3):279–283.		X							
	Alten B, Caglar SS, Kaynas S, Simsek FM. Evaluation of protective efficacy of K-OTAB impregnated bednets for cutaneous leishmaniasis control in Southeast Anatolia-Turkey. J Vector Ecol. 2003;28(1):53–64.		X		X					
	Emami MM, Yazdi M, Guillet P. Efficacy of Olyset long lasting bednets to control transmission of cutaneous leishmaniasis in Iran. East Mediterr Health J. 2009;15(5):1075–83.	X	X				X			
	Jalouk L, Al Ahmed M, Gradoni L, Maroli M. Insecticide-treated bednets to prevent anthroponotic cutaneous leishmaniasis in Aleppo Governorate, Syria: results from two trials. Trans R Soc Trop Med Hyg. 2007;101(4):360–367. Available from: http://www.ncbi.nlm.nih.gov/pubmed/17097698.				X		X			
	Motavalli-Emami M. Impact of Olyset long lasting nets on anthroponotic cutaneous leishmaniasis in Islamic Republic of Iran. WHO Results Portfolio 3 WHO-EM/TDR/110/E. 2006. Available from: http://applications.emro.who.int/dsaf/dsa744.pdf.				X					
	Nadim A, Motabar M, Houshmand B, Keyghobadi K, Aflatonian MR. Evaluation of pyrethroid impregnated bednets for control of anthroponotic cutaneous leishmaniasis in Bam (Islamic Republic of Iran). Geneva: World Health Organization; 1995.		X		X		X			
	Tayeh A, Jalouk L, Al-Khiami AM. A Cutaneous Leishmaniasis Control Trial Using Pyrethroid-Impregnated Bednets in Villages near Aleppo, Syria. WHO WHO/LEISH/. 1997.				X					
	**ITCs (including insecticide-treated house screening)**									
	Kroeger A, Avila EV, Morison L. Insecticide impregnated curtains to control domestic transmission of cutaneous leishmaniasis in Venezuela: cluster randomised trial. BMJ. 2002;325(7368):810–3.	X	X		X		X			
	Noazin S, Shirzadi MR, Kermanizadeh A, Yaghoobi-Ershadi MR, Sharifi I. Effect of large-scale installation of deltamethrin-impregnated screens and curtains in Bam, a major focus of anthroponotic cutaneous leishmaniasis in Iran. Trans R Soc Trop Med Hyg. 2013;107(7):444–450.		X							
	Majori G, Maroli M, Sabatinelli G, Fausto AM. Efficacy of permethrin impregnated curtains against endophilic phlebotomine sandflies in Burkina Faso. Med Vet Entomol. 1989;3(4):441–444.									
	**ITSs and ITFs (including insecticide treated clothing)**									
	Asilian A, Sadeghinia A, Shariati F, Imam Jome M, Ghoddusi A. Efficacy of permethrin-impregnated uniforms in the prevention of cutaneous leishmaniasis in Iranian soldiers. J Clin Pharm Ther. 2003;28(3):175–8.	X			X					
	Soto J, Medina F, Dember N, Berman J. Efficacy of permethrin-impregnated uniforms in the prevention of malaria and leishmaniasis in Colombian soldiers. Clin Infect Dis. 1995;21(3) 599–602.	X			X					
	**Durable wall lining (treated with insecticides) and other measures to protect houses**									
	**EVM**									
	**Mixed studies**									
	Moosa-Kazemi S, Yaghoobi-Ershadi M, Akhavan A, Abdoli H, Zahraei-Ramazani A, Jafari R, et al. Deltamethrin-impregnated bed nets and curtains in an anthroponotic cutaneous leishmaniasis control program in northeastern Iran. Ann Saudi Med. 2007;27(1):6–12.				X					
	Reyburn H, Ashford R, Mohsen M, Hewitt S, Rowland M. A randomized controlled trial of insecticide-treated bednets and chaddars or top sheets, and residual spraying of interior rooms for the prevention of cutaneous leishmaniasis in Kabul, Afghanistan. Trans R Soc Trop Med Hyg. 2000;94(4):361–6.	X	X		X		X			
	Rojas CA, Weigle KA, Tovar R, Morales AL, Alexander B. A multifaceted intervention to prevent American cutaneous leishmaniasis in Colombia: results of a group-randomized trial. Biomedica. 2006;26(Suppl. 1):152–66.	X	X		X					
	**Control of the reservoir host**									
	Ershadi M, Zahraei-Ramazani A, Akhavan A, Jalali-Zand A, Abdoli H, Nadim A. Rodent control operations against zoonotic cutaneous leishmaniasis in rural Iran. Ann Saudi Med. 2005;25(4):309–312.				X					
	**Strengthening vector control operations through health education**									
**VL**	**IRS**									
	Feliciangeli MD, Mazzarri MB, Blas SS, Zerpa O. Control trial of *Lutzomyia longipalpis s*.*l*. in the Island of Margarita, Venezuela. Trop Med Int Health. 2003;8(12):1131–1136.			X						
	Silans PD, Marcelino LN, Dedet J-P, Arias JR. Field monitoring of cypermethrin residual effect on the mortality rates of the phlebotomine sandfly *Lutzomyia longipalpis* in the state of Paraíba, Brazil. Mem Inst Oswaldo Cruz. 1998;93(3):339–344.							X		
	**ITNs (including insecticide-impregnated bednets)**									
	Courtenay O, Gillingwater K, Gomes PA, Garcez LM, Davies CR. Deltamethrin-impregnated bednets reduce human landing rates of sandfly vector *Lutzomyia longipalpis* in Amazon households. Med Vet Entomol. 2007;21(2):168–176.			X						
	Elnaiem DA, Elnahas AM, Aboud MA. Protective efficacy of lambdacyhalothrin-impregnated bednets against *Phlebotomus orientalis*, the vector of visceral leishmaniasis in Sudan. Med Vet Entomol. 1999;13(3):310–314.		X							
	Gidwani K, Picado A, Rijal S, Singh SP, Roy L, Volfova V, et al. Serological markers of sand fly exposure to evaluate insecticidal nets against visceral leishmaniasis in India and Nepal: a cluster-randomized trial. PLoS Negl Trop Dis. 2011;5(9):e1296.						X			
	Picado A, Das ML, Kumar V, Kesari S, Dinesh DS, Roy L, et al. Effect of village-wide use of long-lasting insecticidal nets on visceral Leishmaniasis vectors in India and Nepal: A cluster randomized trial. PLoS Negl Trop Dis. 2010;4(1):e587.		X				X			
	Picado A, Singh SP, Rijal S, Sundar S, Ostyn B, Chappuis F, et al. Longlasting insecticidal nets for prevention of *Leishmania donovani* in India and Nepal: paired cluster randomised trial. BMJ. 2010;341:c6760.	X	X		X		X			
	**ITCs (including insecticide-treated house screening)**									
	Dinesh DS, Das P, Picado A, Davies C, Speybroeck N, Ostyn B, et al. Long-lasting insecticidal nets fail at household level to reduce abundance of sandfly vector *Phlebotomus argentipes* in treated houses in Bihar (India). Trop Med Int Health. 2008;13(7):953–8.	X					X			
	**ITSs and ITFs (including insecticide treated clothing)**									
	**Durable wall lining (treated with insecticides) and other measures to protect houses**									
	Kumar V, Kesari SK, Sinha NK, Palit A, Ranjan A, Kishore K, et al. Field trial of an ecological approach for the control of *Phlebotomus argentipes* using mud & lime plaster. Indian J Med Res. 1995;101:154–156.							X		
	**EVM**									
									
	**Mixed studies**									
	Chowdhury R, Dotson E, Blackstock AJ, McClintock S, Maheswary NP, Faria S, et al. Comparison of insecticide treated nets and indoor residual spraying to control the vector of visceral leishmaniasis in Mymensingh District, Bangladesh. Am J Trop Med Hyg. 2011;84(5):662–7.	X					X			
	Costa CH, Tapety CM, Werneck GL. Control of visceral leishmaniasis in urban areas: randomized factorial intervention trial [Controle da leishmaniose visceral em meio urbano: estudo de intervençao randomizado fatorial]. Rev Soc Bras de Med Trop. 2007;40(4):415–9.	X		X	X					
	Das ML, Banjara M, Chowdhury R, Kumar V, Rijal S, Joshi A, et al. Visceral leishmaniasis on the Indian sub-continent: a multi-centre study of the costs of three interventions for the control of the sandfly vector, *Phlebotomus argentipes*. Ann Trop Med Parasitol. 2008;102(8):729–41.	X								
	Das ML, Roy L, Rijal S, Paudel IS, Picado A, Kroeger A, et al. Comparative study of kala-azar vector control measures in eastern Nepal. Acta Trop. 2010;113(2):162–166.							X		
	de Oliveira SS, de Araujo TM. [Evaluation of control measures for visceral leishmaniasis (kala azar) in an endemic area in Bahia, Brazil (1995–2000)]. Cad Saude Publica. 2003;19(6):1681–1690.			X						
	De Souza VMM, Julião FS, Neves RCS, Magalhães PB, Bisinotto TV, Lima AS, et al. [Communitary assay for assessment of effectiveness of strategies for prevention and control of human visceral leishmaniasis in the municitpality of Feira de Santana, State of Bahia, Brazil]. Epidemiol Serv Saude. 2018;17(2):97–106.			X	X					
	Gavgani ASM, Hodjati MH, Mohite H, Davies CR. Effect of insecticide-impregnated dog collars on incidence of zoonotic visceral leishmaniasis in Iranian children: a matched-cluster randomised trial. Lancet. 2002;360(9330):374–379. Available from: http://www.ncbi.nlm.nih.gov/pubmed/12241778.				X					X
	Joshi AB, Das ML, Akhter S, Chowdhury R, Mondal D, Kumar V, et al. Chemical and environmental vector control as a contribution to the elimination of visceral leishmaniasis on the Indian subcontinent: cluster randomized controlled trials in Bangladesh, India and Nepal. BCM Med. 2009;7:54.	X	X				X	X		
	Kelly DW, Mustafa Z, Dye C. Differential application of lambda-cyhalothrin to control the sandfly *Lutzomyia longipalpis*. Med Vet Entomol. 1997;11(1):13–24.	X								
	Werneck GL, Costa CHN, de Carvalho FAA, Pires e Cruz MdS, Maguire JH, Castro MC. Effectiveness of insecticide spraying and culling of dogs on the incidence of *Leishmania infantum* infection in humans: a cluster randomized trial in Teresina, Brazil. PLoS Negl Trop Dis. 2014;8(10):e3172. doi: 10.1371/journal.pntd.0003172	X					X			
	Romero GAS, Boelaert M. Control of visceral leishmaniasis in Latin America-a systematic review. PLoS Negl Trop Dis. 2010;4(1):e584.					X				
	**Control of the reservoir host**									
	Ashford DA, David JR, Freire M, David R, Sherlock I, Eulálio MC, et al. Studies on control of visceral leishmaniasis: impact of dog control on canine and human visceral leishmaniasis in Jacobina, Bahia, Brazil. Am J Trop Med Hyg. 1998;59(1):53–57.			X	X					
	Braga MD, Coêlho IC, Pompeu MM, Evans TG, MacAullife IT, Teixeira MJ, et al. [Control of canine visceral leishmaniasis: comparison of results from a rapid elimination program of serum-reactive dogs using an immunoenzyme assay and slower elimination of serum-reactive dogs using filter paper elution indirect immunofluorescence]. Rev Soc Bras Med Trop. 1998;31(5):419–424.			X						
	Costa CH. How effective is dog culling in controlling zoonotic visceral leishmaniasis? A critical evaluation of the science, politics and ethics behind this public health policy. Rev Soc Bras Med Trop. 2011;44(2):232–42. doi: 10.1590/S0037-86822011005000014. PubMed PMID: 21468480.					X				
	da Silva V, Borja-Cabrera GP, Correia Pontes NN, de Souza EP, Luz KG, Palatnik M, et al. A phase III trial of efficacy of the FML-vaccine against canine kalaazar in an endemic area of Brazil (São Gonçalo do Amaranto, RN). Vaccine. 2000;19(9–10):1082–1092.			X						
	Dietze R, Barros GB, Teixeira L, Harris J, Michelson K, Falqueto A, et al. Effect of eliminating seropositive canines on the transmission of visceral leishmaniasis in Brazil. Clin Infect Dis. 1997;25(5):1240–1242.			X	X					
	Giffoni JH, de Almeida CE, dos Santos SO, Ortega VS, de Barros AT. Evaluation of 65% permethrin spot-on for prevention of canine visceral leishmaniasis: effect on disease prevalence and the vectors (Diptera: Psychodidae) in a hyperendemic area. Vet Ther. 2002;3(4):485–492.			X						X
	Magalhaes PA, Mayrink W, da Costa CA, Melo MN, Dias M, Batista SM, et al. [Kala-azar in the Rio Doce, Minas Gerais area. Results of prophylactic measures]. Rev Inst Med Trop Sao Paulo. 1980;22(4):197–202.			X						
	Moreira ED Jr, Mendes de Souza VM, Sreenivasan M, Nascimento EG, Pontes de CL. Assessment of an optimized dog-culling program in the dynamics of canine Leishmania transmission. Vet Parasitol. 2004;122(4):245–252.			X						
	Paranhos-Silva M, Nascimento EG, Melro MC, Oliveira GG, dos Santos WL, Pontes-de-Carvalho LC, et al. Cohort study on canine emigration and Leishmania infection in an endemic area for American visceral leishmaniasis. Implications for the disease control. Acta Trop. 1998;69(1):75–83.			X						
	Reithinger R, Coleman PG, Alexander B, Vieira EP, Assis G, Davies CR. Are insecticide-impregnated dog collars a feasible alternative to dog culling as a strategy for controlling canine visceral leishmaniasis in Brazil? Int J Parasitol. 2004;34(1):55–62.			X						
	Aoun K, Chouihi E, Boufaden I, Mahmoud R, Bouratbine A, Bedoui K. Efficacy of deltamethrine-impregnated collars Scalibor in the prevention of canine leishmaniasis in the area of Tunis. Arch Inst Pasteur Tunis. 2008;85(1–4);63–68.									X
	Ferroglio E, Poggi M, Trisciuoglio A. Evaluation of 65% permethrin spot-on and deltamethrin-impregnated collars for canine *Leishmania infantum* infection prevention. Zoonoses Public Health. 2008;55(3):145–148.									X
	Foglia Manzillo V, Oliva G, Pagano A, Manna L, Maroli M, Gradoni L. Deltamethrin-impregnated collars for the control of canine leishmaniasis: evaluation of the protective effect and influence on the clinical outcome of Leishmania infection in kennelled stray dogs. Vet Parasitol. 2006;142(1–2):142–145 (Epub 2006 Aug 2001).									X
	Gomez-Ochoa P, Sabate D, Homedes J, Ferrer L. Clinical efficacy of a Leisguard-based program strategically established for the prevention of canine leishmaniosis in endemic areas with low prevalence. In: 73rd Congresso Internazionale Mutisala SCIVAC. Italy; 2012. p. 545.									X
	Llinas J, Gomez-Ochoa P, Sabate D, Homedes J, Ferrer L. Clinical efficacy of a domperidone-based treatment program for the prevention of canine leishmaniosis. In: South European Veterinary Conference. Barcelona, Spain; 2011.									X
	Maroli M, Mizzoni V, Siragusa C, D’Orazi A, Gradoni L. Evidence for an impact on the incidence of canine leishmaniasis by the mass use of deltamethrin-impregnated dog collars in southern Italy. Med Vet Entomol. 2001;15(4):358–363.									X
	Otranto D, Paradies P, Lia RP, Latrofa MS, Testini G, Cantacessi C, et al. Efficacy of a combination of 10% imidacloprid/50% permethrin for the prevention of leishmaniasis in kennelled dogs in an endemic area. Vet Parasitol. 2007;144(3–4):270–278.									X
	Otranto D, de Caprariis D, Lia RP, Tarallo V, Lorusso V, Testini G, et al. Prevention of endemic canine vector-borne diseases using imidacloprid 10% and permethrin50% in young dogs: a longitudinal field study. Vet Parasitol. 2010;172(3–4):323–332.									X
	Saridomichelakis MN, Mylonakis ME, Leontides LS, Billinis C, Koutinas AF, Galatos AD, et al. Periodic administration of allopurinol is not effective for the prevention of canine leishmaniosis (*Leishmania infantum*) in the endemic areas. Vet Parasitol. 2005;130(3–4):199–205.									X
	**Strengthening vector control operations through health education**									
	Isaza DM, Restrepo BN, Arboleda M, Casas E, Hinestroza H, Yurgaqui T. La leishmaniasis: Conocimientos y prácticas en poblaciones de la costa del Pacifico de Colombia. Rev Panam Salud Públ. 1999;6(3):177–184.								X	
	da Luz ZMP, Schall V, Rabello A. Evaluation of a pamphlet on visceral leishmaniasis as a tool for providing disease information to healthcare professionals and laypersons. Cad Saúde Pública. 2005;21(2):606–621.								X	
	Magalhães DF, da Silva JA, Haddad JPA, Moreira EC, Fonseca MIM, de Ornelas ML, et al. Dissemination of information on visceral leishmaniasis from schoolchildren to their families: a sustainable model for controlling the disease. Cad Saúde Pública. 2009;25(7):1642–1646.								X	
	Reis DC, Gazzinelli A, Silva CAB, Gazzinelli MF. Educação em saúde e representações sociais: uma experiência no controle da leishmaniose tegumentar em área endêmica de Minas Gerais, Brasil. Cad Saúde Pública. 2006;22(11):2301–2310.								X	
	Uchôa CMA, Serra CMB, Magalhães CM, da Silva RMM, Figliuolo LP, Leal CA, et al. Educação em saúde: ensinando sobre a leishmaniose tegumentar americana. Cad Saúde Pública. 2004;20(4):935–941.								X	
	**Descriptive studies**									
CL	Armijos RX, Weigel MM, Izurieta R, Racines J, Zurita C, Herrera W, et al. The epidemiology of cutaneous leishmaniasis in subtropical Ecuador. Trop Med Int Health. 1997;2(2):140–152.							X		
	Bauer IL. Knowledge and behavior of tourists to Manu National Park, Peru, in relation to leishmaniasis. J Travel Med. 2002;9(4):173–179.									
	Bsrat A, Berhe N, Balkew M, Yohannes M, Teklu T, Gadisa E, et al. Epidemiological study of cutaneous leishmaniasis in Saesie Tsaeda-emba district, eastern Tigray, northern Ethiopia. Parasit Vectors. 2015;8:149.							X		
	de Castro EA, Luz E, Telles FQ, Pandey A, Biseto A, Dinaiski M, et al. Eco-epidemiological survey of *Leishmania (Viannia) braziliensis* American cutaneous and mucocutaneous leishmaniasis in Ribeira Valley River, Paraná State, Brazil. Acta Trop. 2005;93(2):141–149.							X		
	Pedrosa Fde A, Ximenes RA. Sociodemographic and environmental risk factors for American cutaneous leishmaniasis (ACL) in the state of Alagoas, Brazil. Am J Trop Med Hyg. 2009;81(2):195–201.							X		
	Reithinger R, Mohsen M, Leslie T. Risk factors for anthroponotic cutaneous leishmaniasis at the household level in Kabul, Afghanistan. PLoS Negl Trop Dis. 2010;4(3):e639.							X		
	Rodriguez-Villamizar LA, Orozco-Vargas LC, Muñoz-Mantilla G. The basic health plan’s impact on preventing cutaneous leishmaniasis in rural areas of Santander, Colombia [article in Spanish]. Rev Salud Publica (Bogota). 2006;8(Suppl 1):116–128.							X		
	Votypka JI, Kasap OE, Volf P, Kodym P, Alten B. Risk factors for cutaneous leishmaniasis in Ukurowa region, Turkey. Trans R Soc Trop Med Hyg. 2012;106(3):186–190.							X		
	Yadon ZE, Rodrigues LC, Davies CR, Quigley MA. Indoor and peridomestic transmission of American cutaneous leishmaniasis in northwestern Argentina: a retrospective case control study. Am J Trop Med Hyg. 2003;68(5):519–526.							X		
VL	Barnett PG, Singh SP, Bern C, Hightower AW, Sundar S. Virgin soil: the spread of visceral leishmaniasis into Uttar Pradesh, India. Am J Trop Med Hyg. 2005;73(4):720–725.							X		
	Boelaert M, Meheus F, Sanchez A, Singh SP, Vanlerberghe V, Picado A, et al. The poorest of the poor: a poverty appraisal of households affected by visceral leishmaniasis in Bihar, India. Trop Med Int Health. 2009;14(6):639–644.							X		
	Bern C, Joshi AB, Jha SN, Das ML, Hightower A, Thakur GD, et al. Factors associated with visceral leishmaniasis in Nepal: bed-net use is strongly protective. Am J Trop Med Hyg. 2000;63(3–4):184–188.							X		
	Costa CH, Werneck GL, Rodrigues L, Santos MV, Araújo IB, Moura LS, et al. Household structure and urban services: neglected targets in the control of visceral leishmaniasis. Ann Trop Med Parasitol. 2005;99(3):229–236.							X		
	Kesari S, Bhunia GS, Kumar V, Jeyaram A, Ranjan A, Das P. Study of house-level risk factors associated in the transmission of Indian kala-azar. Parasit Vectors. 2010;3:94.							X		
	Nackers F, Mueller YK, Salih N, Elhag MS, Elbadawi ME, Hammam O, et al. Determinants of visceral leishmaniasis: a case-control study in Gedaref state, Sudan. PLoS Negl Trop Dis. 2015;9(11):e0004187.							X		
	Perry D, Dixon K, Garlapati R, Gendernalik A, Poché D, Poché R. Visceral leishmaniasis prevalence and associated risk factors in the saran district of Bihar, India, from 2009 to July of 2011. Am J Trop Med Hyg. 2013;88(4):778–784.							X		
	Quinnell RJ, Dye C. An experimental study of the peridomestic distribution of *Lutzomyia longipalpis* (Diptera: Psychodidae). Bull Ent Res. 1994;84(3):379–382.							X		
	Ranjan A, Sur D, Singh VP, Siddique NA, Manna B, Lal CS, et al. Risk factors for Indian kala-azar. Am J Trop Med Hyg. 2005;73(1): 74–78.							X		
	Ryan JR, Mbui J, Rashid JR, Wasunna MK, Kirigi G, Magiri C, et al. Spatial clustering and epidemiological aspects of visceral leishmaniasis in two endemic villages, Baringo district, Kenya. Am J Trop Med Hyg. 2006;74(2):308–317.							X		
	Saha S, Ramachandran R, Hutin YJF, Gupte MD. Visceral leishmaniasis is preventable in a highly endemic village in West Bengal, India. Trans R Soc Trop Med Hyg. 2009;103(7):737–742.							X		
	Schaefer KU, Kurtzhals JA, Kager PA, Gachihi GS, Gramiccia M, Kagai JM, et al. Studies on the prevalence of leishmanin skin test positivity in the Baringo district, rift valley, Kenya. Am J Trop Med Hyg. 1994;50(1):78–84.							X		
	Schenkel K, Rijal S, Koirala S, Koirala S, Vanlerberghe V, Van der Stuyft P, et al. Visceral leishmaniasis in southeastern Nepal: a cross-sectional survey on *Leishmania donovani* infection and its risk factors. Trop Med Int Health. 2006;11(12):1792–1799.							X		
	Singh SP, Hasker E, Picado A, Gidwani K, Malaviya P, Singh RP, et al. Risk factors for visceral leishmaniasis in India: further evidence on the role of domestic animals. Trop Med Int Health. 2010;15(Suppl 2):29–35.							X		
	Uranw S, Hasker E, Roy L, Meheus F, Das ML, Bhattarai NR, et al. An outbreak investigation of visceral leishmaniasis among residents of Dharan town, eastern Nepal, evidence for urban transmission of Leishmania donovani. BMC Infect Dis. 2013;13:21.							X		
	Yared S, Deribe K, Gebreselassie A, Lemma W, Akililu E, Kirstein OD, et al. Risk factors of visceral leishmaniasis: a case control study in north-western Ethiopia. Parasit Vectors. 2014;7:470.							X		

CL, cutaneous leishmaniasis; EVM, environmental modification; IRS, indoor residual spraying; ITC, insecticide-treated curtain; ITF, insecticide-treated fabric; ITM, insecticide-treated material; ITN, insecticide-treated net; ITS, insecticide-treated bedsheet; NTD, neglected tropical disease; SR, systematic review; VL, visceral leishmaniasis.

### Type of studies included in the SRs/MAs

The SRs/MAs included different types of primary studies, if categorising with the hierarchy of evidence (https://consumers.cochrane.org/levels-evidence). Some SRs/MAs used randomised controlled trials (RCTs) and/or cluster randomised controlled trials (cRCTs) only [14,16–18], and others used studies with lower level of evidence as well [12,13,15,19,20]. There was some overlap of the included studies, particularly for the RCTs (see Table 3). However, Table 3 clearly indicates that the different SRs/MAs used substantially different studies, with a total of 88 articles included in 9 SRs/MAs.

### Type of outcome measures

Human disease parameters were mostly not captured in the studies included in the SRs/MAs. However, one SR focused on studies with human disease parameters [14], and another SR [15] assessed studies that reported, beside entomological parameters, clinical data focusing on CL and VL incidence in intervention and control groups.

Entomological parameters applied varied considerably. In some studies, the entomological parameters applied were of vector density or mean number of phlebotomine sand flies (per house per night) between intervention and control groups either using IRS, ITNs, or insecticide-treated curtains (ITCs) [12,15–18,20]. Other entomological parameters were identified such as sand fly landing rates and vector mortality rates [12,20].

SRs that included studies on reservoir control applied canine VL incidence and prevalence parameters [12,16,19].

### Quality analysis

Quality analysis showed that, when measuring against the PRISMA criteria [10], several SRs/MAs meet most or all quality criteria, above 24 or all of 27 criteria [15,18,19]. Further three SRs meet between 20 and 23 criteria [13,17,20]. And 3 SRs met only 16 or below criteria.

### Analysis of results

#### Crosscutting results

The methods analysed in the SRs/MAs for controlling vectors to prevent transmission of the parasite causing either CL or VL have been found to be similar. The same method or technique has been named slightly differently by groups of researchers in different settings. The methods included IRS, the use of ITNs (including insecticide-impregnated bednets), ITCs (including insecticide-treated house screening), insecticide-treated bedsheets (ITSs), and insecticide-treated fabrics (ITFs) (including insecticide-treated clothing) and durable wall lining (treated with insecticides) and other measures to protect houses. In addition, environmental modifications (EVMs), control of the reservoir host, and strengthening vector control operations through health education were evaluated. Many of the studies included in the SRs/MAs evaluated several interventions at the same time.

This main results section presents the results therefore as

1. Results for CL only;
2. Results for VL only;
3. Results for CL and VL;
4. Results for controlling the reservoir host;
5. Results for control through education; and
6. Gap analysis.

#### Results for CL only

*IRS*. For IRS in the context of studies looking at CL only, González and colleagues (2015) [17] and Stockdale and Newton (2013) [14] reported mixed results: Only 2 cRCTs from South America were included by González and colleagues (2015), with substantial reductions of vectors, without evidence for long-term effects. An additional cRCT from Afghanistan reported a reduction of human CL cases over 15 months. Stockdale and Newton (2013) [14], however, included 4 studies, limiting the studies to those presenting human measurements, and presenting mixed results, with 2 higher quality studies showing no efficacy of IRS, whereas 2 lower quality studies showed some efficacy in reduction of cases.

*ITNs*. For ITNs in relation to CL, 3 SRs/MAs conducted an analysis [14,15,17], with more positive results, both on vectors and human transmission: González and colleagues (2015) [17] analysed 3 studies testing ITNs versus no intervention or untreated nets on CL. One of 3 cRCTs in Iran evaluated the effect of ITNs on vector density, with a statistically significant reduction, however not measuring duration of effect. Two cRCTs from Afghanistan and Iran measured incidence of CL, with a marked reduction of CL cases over 15 months. Wilson and colleagues (2014) [15] with its focus on ITNs, included 6 studies evaluating the efficacy of ITNs against CL, allowing also for an MA. Random effect MA indicated a partial effect of 77%. However, studies assessing the efficacy of ITNs reported mixed results in terms of effect on vector density, ranging from a relative increase of 49% to a relative reduction of 96%. Stockdale and Newton (2013) [14] reported on 5 studies with a statistically significant reduction of cases of CL, however underlining that for 4 of the included studies, self-reporting was used for case definition.

*ITCs*. Also, ITCs were evaluated by 3 SRs/MAs [14,15,17].

Reduction of vector indices varied; however, reduction of human transmission indices was positive: González and colleagues (2015) [17] included 1 cRCT, although no statistically significant differences in the mean number of vectors were reported, the incidence of clinical cases of CL were 0/1,351 (0%) in the intervention group and 142/1,587 (9%) in the control group. Wilson and colleagues (2014) [15] reported a high percentage reduction in vector density of 54%, 87%, and 98% for the included studies; however, the 98% reduction was observed in a study that was deemed to be of low quality. Stockdale and Newton (2013) [14] reported only for one of 4 studies using ITCs a statistically significant decrease of CL cases.

*ITSs and ITFs*. ITSs/ITFs were again evaluated by 3 SRs/MAs [14,15,17], with overall positive effects on human transmission. González and colleagues (2015) [17] analysed ITS (bedsheets) versus no intervention on CL with a cRCT from Afghanistan, with substantially fewer cases over 15 months in the intervention households across all age groups. Two RCTs evaluated the effect of impregnating soldiers’ uniforms with permethrin on the incidence of CL. The trials were small and underpowered to confidently detect or exclude effects. However, in one study, the incidence in the control group was 18/143 over 12 weeks (12%), and just 4/143 (3%) in soldiers with impregnated uniforms (risk ratio (RR) 0.22, 95% CI 0.08 to 0.64). Stockdale and Newton (2013) [14] included 4 studies with ITFs, and three reported a statistically significant decrease in numbers of human cases of CL between intervention and control groups.

*EVM*. Only Stockdale and Newton (2013) [14] analysed EVM against CL; none of the outcomes of the included studies were measured against human transmission indicators.

#### Results for VL only

Vector control methods analysed in the articles were essentially the same as for CL, and the same categories were used for the analysis.

*IRS*. IRS was analysed by González and colleagues (2015) [17], Romero and Boelaert (2010) [12], and Stockdale and Newton (2013) [14] in the context of VL.

No trials evaluated the effects of IRS on VL incidence, as stated by González and colleagues (2015) [17]. However, one trial assessed the effect on seroconversion in a VL endemic area in Brazil and found no statistically significant difference in seroconversion over 18 months post-intervention. Romero and Boelaert (2010) [12] however reported evaluating IRS and fogging around the houses, a significant decrease of sand fly abundance, with a residual effect of indoor spraying, which usually lasted 3 months which was influenced mainly by house construction style. Stockdale and Newton (2013) [14] included 2 studies with no trend of effect: one evaluated VL in children under 12 years in 3 control and 3 intervention areas, with no difference in infections rates in all the evaluated areas. The other study used random allocation of 4 intervened areas in order to study VL in the population living in each area; the study observed no difference between the intervention and control areas.

*ITNs*. ITNs were analysed by González and colleagues (2015) [17], Romero and Boelaert (2010) [12], and Wilson and colleagues (2014) [15]. González and colleagues (2015) [17] reported on ITNs versus no intervention or untreated nets on VL. Two of the 3 included cRCTs in Asia evaluated the effect of ITNs on vector density: In Bangladesh, there was a substantial reduction in vector density in the ITN areas for 12 months post-intervention, but in a multicentre trial in Asia, the overall difference between intervention and control sites was not statistically significant. One additional cRCT in India reported a statistically significant reduction in male *P*. *argentipes* in areas with ITNs compared to untreated nets, but no difference in female *P*. *argentipes* or other vectors.

One cRCT evaluated the effect of ITNs on VL in India and Nepal. The overall risk of VL during the 30 months follow-up was 37/9,829 (0.38%) in the intervention group and 40/9,981 (0.40%) in the control group. In the same trial, there was also no significant difference in the risk of seroconversion in those who had negative results at baseline [17].

Wilson and colleagues (2014) [15] also compared the efficacy of ITNs against VL, including 3 studies. Similarly, one study did not show a significant effect on incident *Leishmania donovani* infections or incident cases of VL. However, in India and Nepal, the same study did appear to show an effect on vector density with a relative reduction in the mean number of female *P*. *argentipes*. Two studies conducted in Sudan and Bangladesh, India, and Nepal demonstrated a 100% and 35% reduction in vector density, respectively.

Romero and Boelaert (2010) [12] only included 1 study, with ITNs and VL: The study described a 39% increase in barrier capacity of the deltamethrin-impregnated bednets, 80% reduction in sand fly landing rates on humans, and 98% increase in the 24-hour sandfly mortality rates. The study had many limitations (a small number of observations, a short period of exposure, and without the measure of the residual effect).

Stockdale and Newton (2013) [14] reported as well on CL and VL in relation to ITN, with no clear effect reported (see the relevant section below).

*ITCs*. For ITCs and VL, no studies were included in the SRs/MAs.

*ITSs and ITFs*. Only González and colleagues (2015) [17] included studies on ITSs: one cRCT in areas of Brazil with VL evaluated the effects of treated sheets near the chicken shed, with short-term reductions in geometric mean phlebotomine sand flies per trap after the intervention, which only differed statistically from control sheds at week 12 post-intervention.

*EVM*. González and colleagues (2015) [17] included 2 cRCTs, comparing EVM versus no intervention: Neither trial found evidence of statistically significant reductions in phlebotomine sand flies compared to no intervention up to 12 months follow-up.

EVM has been further analysed in the context of both CL and VL (see the relevant section below).

#### Results for CL and VL

The included SRs/MAs also compared studies with information on both CL and VL.

*IRS*. González and colleagues (2015) [17] included studies on IRS versus no intervention, with 2 included cRCTs, reporting substantial reductions in vectors at the intervention sites. Calderon-Anyosa and colleagues (2018) [20] reported on 8 intervention studies describing housing characteristics, sand fly density captured by light traps (5/8), and sand fly mortality by wall bioassay (3/8). Of the 8 studies, 4 evaluated the effect of insecticidal spray on different wall materials. One evaluated sand fly density and reported differences associated with housing quality and vector densities; in some cases, the number was higher even after insecticide thermal fogging.

From the remaining studies that evaluated wall bioassay mortality, one evaluated fogging on cement wall versus oil-painted wall, finding no significant differences in sand fly mortality at 7 or 125 days after fogging, whereas it was significantly higher in oil-painted wall at 69 days. The other study matched houses according to their structure and were randomly assigned to spray treatment or control, finding that sand fly mortality decreased progressively on wood and cement surfaces after 63 days compared with a more rapid decrease on mud and straw walls. The third study evaluated spray on the external and internal surfaces of 3 types of walls, finding that mortality rates were similar, whatever the type of wall, since the fourth month.

*ITNs*. ITNs were analysed again by Stockdale and Newton (2013) [14]: For human reservoir control, 7 studies using ITNs measured a human-specific outcome. Two studies used deltamethrin-impregnated bednets. Neither group reported any difference in cases of CL or VL between the treated nets and either untreated nets or existing intervention.

*ITSs and ITFs*. No studies on ITSs/ITFs were included.

*EVM*. EVM was included by Horstick and Runge-Ranzinger (2018) [18], concluding that modifications to the structure of houses (e.g., wall plastering) had no impact on the control of vectors. However, protection of the house and its surroundings might affect the transmission of several diseases.

Calderon-Anyosa and colleagues (2018) [20] described housing characteristics and risk for presence of vectors and disease: Mud walls with cracks and holes, damp, and dark houses were risk factors for transmission of leishmaniasis. These characteristics create favourable conditions for sand fly breeding and resting as sand flies prefer humidity, warmth, and protection from sunlight during the day. A total of 18/23 studies found significant association between housing characteristics (e.g., walls, roof, floors, or windows) and leishmaniasis infection or sand fly density. Moreover, 16/18 studies found an association between leishmaniasis and wall type. A total of 15/16 studies found an association with clinical leishmaniasis: 5/15 with CL cases and 10/15 with VL cases. In addition, 4/8 intervention studies evaluated housing characteristics and home improvement against sand fly density captured by light traps. One experimental study evaluated the characteristics of chicken sheds against sand fly densities and found a significantly higher number of sand flies in open sheds. The 3 remaining studies evaluated the effect of plastering and closing crevices against sand fly densities: One study found no significant difference in sand fly density, whereas the other two found a decrease in sand fly density after the intervention.

#### Results for controlling the reservoir host

One SR [19] focused exclusively on the control of the reservoir host assessing the following studies: the use of insecticide-treated dog collars (4 studies including non-RCTs and a matched-cluster RCT) and a combination of dog collars and spot-on insecticides treatments (1 non-RCT). There was a statistically significant protective effect of collars, measured by the overall proportion of dogs infected with *Leishmania infantum*. Use of spot-on insecticides treatments (3 studies, including non-RCTs and RCTs): there was a statistically significant protective effect for the overall proportion of dogs infected with *L*. *infantum*. Three studies (all RCTs) evaluated prophylactic medications: 2 studies for domperidone liquid solution and 1 study for allopurinol capsules. There was a statistically significant protective effect for prophylactic medication with domperidone for the overall proportion of dogs infected with *L*. *infantum*, but not for allopurinol.

#### Results for control through education

Furthermore, education was the focus of one SR, in South America [13]. Five studies evaluated the influence of educational material showing an improvement or reinforcing the importance of educational activities to improve access to knowledge by the population.

One study showed the actions of local social representations as effective instruments of information and prevention of leishmaniasis. Also, including guidance to the public on the use of screens and mosquito nets impregnated with insecticide was evaluated. One study found that although 94% of participants knew leishmaniasis as a skin disease, with ulcers or blemishes, only 35% associated the disease with the bite of an infected “mosquito,” and only 10% used the appropriate drug treatment.

### Gap analysis

#### Gap analysis of summary evidence of vector control

When analysing the existent SRs/MAs for both CL and VL, and including studies to control the reservoir host by vector control measures, it is positive to note that there are 9 SRs, with 1 SR also presenting MA data [15] (Table 1).

However, all SRs/MAs have a different research question and inclusion/exclusion criteria differ as well (Table 1). Assessing the quality of the SRs/MAs, there is a considerable degree of variation, with a trend of later published SRs achieving better quality (Table 1). Similarly, the SRs were all published after 2010, with the latest published in 2018 (Table 1). This reflects inclusion of studies published earlier than 2018, with data collected even earlier.

Hence, even for similar questions, for example, efficacy and community effectiveness of IRS in the context of CL, the SRs dealing with this question include different studies (Table 3). And the information used may be outdated. With this approach, it is difficult to find agreement for policy recommendations (see Box 1 for a summary).

**Box 1. Summary of key results**

**CL**

IRS: inconclusive results, on both vectors and human transmission indicators

ITNs: some agreement of reduction of both vectors and human transmission indicators

ITCs: inconclusive results for vectors, but reduction of clinical cases

ITSs and ITFs: overall positive effect on human transmission indicators

EVM: Poor level of evidence, no clear effect

**VL**

IRS: inconclusive results, with reduction of vectors, but only little available evidence for reduction of cases

ITNs: some agreement of reduction of vectors, with a negative effect reported as well, but no clear reduction of human transmission indicators

ITCs: no studies

ITSs and ITFs: poor level of evidence and no clear effect on vectors

EVM: no reduction of vectors

**CL/VL**

No clear additional information for those studies looking at both CL and VL

IRS: more positive

ITN: no clear results

ITSs/ITFs: not analysed

EVM: not clear

**Control of reservoir host**

Insecticide-treated collars, spot-on insecticides, and medication with domperidone seem to have positive effects on reservoir host infection. In addition, dog culling as a control intervention was found to be consistently ineffective.

#### Education

It is difficult to assess the effect of education on transmission with the included studies; however, a relation between knowledge of disease transmission and protective behaviour is assumed.

## Discussion

### Discussion of key results

Vector control for CL and VL has been targeted using different tools in different settings at different times, as shown with the multiple studies included in the SRs/MAs. The methods used for controlling vectors to prevent transmission of the parasite causing either CL or VL have been found to be similar, including IRS, ITNs—mostly insecticide-impregnated bednets, ITCs, ITSs and ITFs, and durable wall lining (treated with insecticides) and other environmental measures to protect houses.

The key results are very difficult to interpret with a lot of contradictory messages, and it is difficult to describe a clear trend. The SRs/MAs that we included in our meta-review identified gaps in control measures, especially in relation to their evaluation rather than implementation. Given the SRs/MAs could only include the original research findings available up to the time point of their literature search, these reviews could also omit more recent investigations on CL and VL vector control. This is more likely to happen especially for the recent past when the control measures were strengthened with more technical cooperation between the countries, along with revised VL elimination targets for South Asia and the region [5]. Also, new vector control techniques could have been missed in the SRs/MAs. For other regions, because of the zoonotic nature of the disease, especially for VL, the reviews and investigations were split based on their focus on either humans or animals. This made the gap analysis from our meta-review constrained as we had less focus on vector control in animal reservoirs.

The gaps in the research findings identified through our meta-review have been discussed below under themes focusing on diseases as well as vector control methods, techniques, or tools. Also, there are overarching issues around control of the vectors irrespective of what disease they are causing—CL or VL. We combine below those overarching issues and individual disease specific considerations while discussing gaps in findings.

In majority of the studies included in different SRs/MAs, human disease was not considered as the primary outcome of interest. This clearly left a gap in understanding the associations between different vector control measures and their eventual impact in reducing burden of CL or VL in humans. Moreover, in the studies which used human disease as their outcome, case identification often relied on clinical symptoms, patient reporting, antibody detection, and clinical cure. Parasite detection through their visualisation was rarely performed, which could have resulted in misclassifications of disease condition, especially for VL which could mimic other endemic conditions in the study areas and regions [21,22]. Depending on the nature of bias, this could result in underestimation or overestimation of the associations between vector control measures and the occurrence of the disease. Some studies also lacked adequate power to detect a true association. All these could have affected the internal validity of the studies performed by different groups of researchers in different settings and regions. Generalisability of the study findings to all settings was also difficult because of this as well as due to the zoonotic and anthroponotic divide of the nature of VL by regions.

Moreover, research methodological variations (interventional versus observational) and weaknesses also made the studies less similar and difficult to summary effect measure estimation. The methodological weaknesses identified by the meta-review include issues around randomisation, blinding, inadequate sample size, participant adherence to the interventions, varying follow-up period, lack of adjustment of possible confounders through multivariable analysis, lack of adjustment of clustering, and poor description of trial designs. This is also reflected in the relative variation of the quality assessment of the included SRs/MAs. Implementation of intervention was also found problematic, for example with IRS not occurring at the same time for all study areas under the intervention arm with equal frequencies and different insecticides were used in different studies, especially in South Asia. Variation in insecticide susceptibility of the sand fly vector could also be problematic [23]. Also, control groups were poorly defined in some studies, whereas some studies had intervention and control arms not comparable due to their varying background VL prevalence.

The vector nature including species and their preferences for hosts [24] as well as resting and feeding indoor (endophagic and endophilic) or outdoor (exophagic and exophilic) needed to be accounted for in a more systematic way to better understand the impact of different interventions. The flying pattern of sand flies could also have been investigated more, especially when looking at the association between house structure and leishmaniasis. Data on intervention studies on house structure and leishmaniasis were also scarce, although more recent observational data have become available [25].

The studies considered in different SRs/MAs were mostly done in controlled environments without takings contexts into account. Further studies were needed to assess the implementation or operational aspect of vector control measures [9]. This might have been done in the recent past, especially in South Asia when they were reaching closer to the elimination target. But given no SR was conducted recently with a single focus on leishmaniasis, those studies might have been missed. Also, since no SR/MA assessed single vector control method only, reviewing each single vector control method including the new ones is warranted to identify more robust evidence on their efficacy and community effectiveness [26].

### Discussion of level of evidence

Policy recommendations should be evidence based [11] and following a systematic approach of weighing and grading available evidence, including a process of expert consensus.

In the context of CL and VL, there is a wealth of available studies, primary studies, and including summary evidence, one of the key results of this meta-review. The existing SRs/MAs include a large variation of different studies, related to the different research question of each individual SR/MA, and the different inclusion/exclusion criteria.

However, as presented in the gap analysis of the results section, it is very difficult to summarise the results of the available SRs/MAs, with the shortcomings described above and it is difficult to recommend with the currently existing SRs/MAs particular vector control methods, or combinations of vector control methods.

A process should be initialised to systematically assess all available evidence for efficacy and community effectiveness of vector control in the context of both CL and VL.

One of the options, mentioned above, would be to assess each single vector control method with a specific SR, and if possible MA. This concept has the advantage to include more studies, on different levels of hierarchy, and to assess—if the information is available—different levels of transmission scenarios.

These SRs should encompass the key vector control methods, e.g.,

1. indoor residual spraying (IRS);
2. ITNs (including insecticide-impregnated bednets);
3. ITCs (including insecticide-treated house screening) and ITSs;
4. ITFs (including insecticide-treated clothing) and durable wall lining (treated with insecticides) and other measures to protect houses;
5. EVMs.
  Technically, it is recommendable to assess these methods for the 2 diseases separately.
  Further specific SRs could be
6. vector control methods of the reservoir host in the context of VL;
7. strengthening vector control operations through health education; and
8. implementation of vector control programmes taking local and regional contexts into account.

With this process, it is expected that a better policy recommendation can be formulated, following a discussion of the results of the SRs/MAs to be developed, in expert consensus.

## Conclusions

This meta-review has answered 3 key objectives:

1. Establishing what is known about the value of vector control for the control of CL and VL: Unfortunately, a clear trend for efficacy and community effectiveness of the different vector control methods for CL and VL is difficult to assess by the existing SRs/MAs. This is mostly due to the different research questions and studies included in each SR/MA. Considering this fact, it is not easy to formulate evidence-based recommendations for vector control methods for CL and VL.
2. Establishing gaps in knowledge: Clearly, there is a wealth of primary studies available to assess vector control for both CL and VL. Further specific gaps for primary research may emerge through a more thorough analysis of each vector control methods. Additionally, there is a gap of systematic assessment of each vector control method.
3. Key recommendations for further scientific work: To improve policy recommendations, one of the key elements for further scientific work is a systematic analysis of each individual vector control methods, e.g., IRS, ITNs, ITCs, ITFs, and EVM, for CL and VL separately, including vector and human transmission parameters and attempting to conclude with recommendations in different transmission scenarios. It may be of interest to conduct SRs/MAs on reservoir host control, education, and programme implementation in support of vector control operations.

**Key learning points**

- A clear trend for efficacy and community effectiveness of the different vector control methods for cutaneous leishmaniasis (CL) and visceral leishmaniasis (VL) is difficult to assess by the existing systematic reviews and meta-analyses (SRs/MAs).
- There is a need to develop further systematic analysis of each individual vector control methods for CL and VL separately.
- The control of reservoir host through insecticide-treated collars, spot-on insecticides, and medication with domperidone seems to have positive effects on reservoir host infection.
- There is no clear effect of education on CL and VL transmission with the included studies.

**Top five papers**

1. González U, Pinart M, Sinclair D, Firooz A, Enk C, Vélez ID, et al. Vector and reservoir control for preventing leishmaniasis. Cochrane Database Syst Rev. 2015 Aug 5;(8):CD008736.
2. Wilson AL, Dhiman RC, Kitron U, Scott TW, Berg H van den, Lindsay SW. Benefit of Insecticide-Treated Nets, Curtains and Screening on Vector Borne Diseases, Excluding Malaria: A Systematic Review and Meta-analysis. PLoS Negl Trop Dis. 2014 Oct 9;8(10):e3228.
3. Romero GAS, Boelaert M. Control of visceral leishmaniasis in latin america-a systematic review. PLoS Negl Trop Dis. 2010 Jan 19;4(1):e584.
4. Stockdale L, Newton R. A Review of Preventative Methods against Human Leishmaniasis Infection. PLoS Negl Trop Dis. 2013 Jun 20;7(6):e2278.
5. Kappagoda S, Ioannidis JPA. Prevention and control of neglected tropical diseases: overview of randomized trials, systematic reviews and meta-analyses. Bull World Health Organ. 2014 May 1;92(5):356-366C.
